# Boron‐Formazanate Complexes as Tunable Redox‐Active Materials for Non‐Aqueous Redox Flow Batteries

**DOI:** 10.1002/chem.202503592

**Published:** 2026-01-05

**Authors:** Reinder H. Bouma, Mitchell J. Demchuk, Suhjung Chun, Francis L. Buguis, Erin L. Cotterill, Arvin M. Mehdian, Paul D. Boyle, Marcus W. Drover, Joe B. Gilroy, Edwin Otten

**Affiliations:** ^1^ Stratingh Institute for Chemistry University of Groningen Groningen The Netherlands; ^2^ Department of Chemistry The University of Western Ontario London Canada

**Keywords:** bipolar electrochemistry, boron, formazanate, heterocycle, main group elements, redox flow battery

## Abstract

As a broad‐scale energy storage solution, redox flow batteries (RFBs) offer high efficiency and tunable design. However, conventional RFBs rely on transition‐metal ion couples, (e.g., vanadium or iron), whose implementation is limited by low energy densities, high cost, and environmental leaching. Main‐group compounds, comprising earth‐abundant, *p*‐block elements, represent highly promising, yet underexplored candidates for RFBs. Herein, we evaluate three boron‐formazanate complexes as negolyte and symmetric electrolytes in nonaqueous organic redox flow batteries (NAORFBs). Detailed electrochemical characterization of these complexes reveals two sequential reduction processes with the first being exceptionally stable (<3% capacity fade after charge/discharge cycling in a static H‐cell for 3 days). In contrast, cycling that includes the two‐electron reduced state results in rapid degradation (>59% capacity fade over 2.5 days in a static H‐cell), most likely due to fluoride elimination from the BF_2_ moiety. Guided by these insights, a B(Ph)_2_ unit was introduced to mitigate this degradation pathway. The elimination of labile B─F bonds as well as steric protection conferred by two phenyl groups led to improved cycling performance (>85% capacity retention after charge/discharge cycling in a flow battery for 15 days). These findings guide the rational design of inexpensive main‐group electrolytes for application in energy storage.

## Graphical abstract

Boron‐formazanates were investigated as a chemically tunable and electrochemically robust class of redox‐active materials for nonaqueous flow batteries. Electrochemical characterization, coupled with postmortem analysis, uncovers B─F bond cleavage as a key degradation pathway contributing to capacity fade. Through rational design, a diphenyl boron derivative was developed that exhibited superior cycling stability in a symmetrical flow cell.

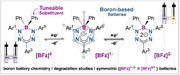

## 1 Introduction

Achieving climate neutrality requires a shift toward renewable energy solutions [1, 2]. However, the intermittent nature of wind and solar power has created the need for large‐scale energy storage [3, 4, 5]. Redox flow batteries (RFBs) have emerged as a promising technology due to their high efficiency and flexible design, through independent scaling of power output and total storage capacity [6, 7, 8]. Conventional RFBs typically rely on transition‐metal ion couples (e.g., of vanadium) in aqueous electrolytes [9, 10]. While these systems have demonstrated excellent cycling stability, their implementation is limited by low energy densities, relatively high cost, and environmental concerns [11, 12].

In response to these limitations, growing interest has been directed toward the study of nonaqueous organic redox flow batteries (NAORFBs) [13]. These systems utilize organic solvents, which have a significantly wider electrochemical window than water and thereby offer the potential to achieve higher cell voltages and energy densities [14, 15]. Organic redox active materials are promising due to their earth‐abundant composition, cost‐effectiveness, and synthetic accessibility from noncritical raw materials [16, 17, 18, 19]. Furthermore, their molecular structure allows for chemical modification to tune redox potential, solubility, and overall battery performance [14, 20].

Despite these advantages, small organic molecules often suffer from inadequate long‐term cycling stability, which remains a limiting factor to their widespread utilization in RFBs [21, 22, 23]. Transition metal coordination compounds are also used as redox‐active materials, as these systems combine the molecular tunability of organics with the potentially enhanced stability offered by coordination environments—features that are inherent to many main group organometallics [24, 25, 26].

In comparison to organic and transition metal compounds, molecules containing main group elements as part of the redox‐active unit have not received much attention in the context of flow battery materials. Nevertheless, recent reports on phosphine oxide‐containing compounds (as a representative class of main group redox‐active species) indicate that these can have extreme redox potentials as well as promising stability [27, 28]. Of the *p*‐block elements, boron is a versatile center for the development of redox‐active materials, both in simple compounds with a single B atom as well as in B‐based clusters [29, 30, 31, 32].

**FIGURE 1:**
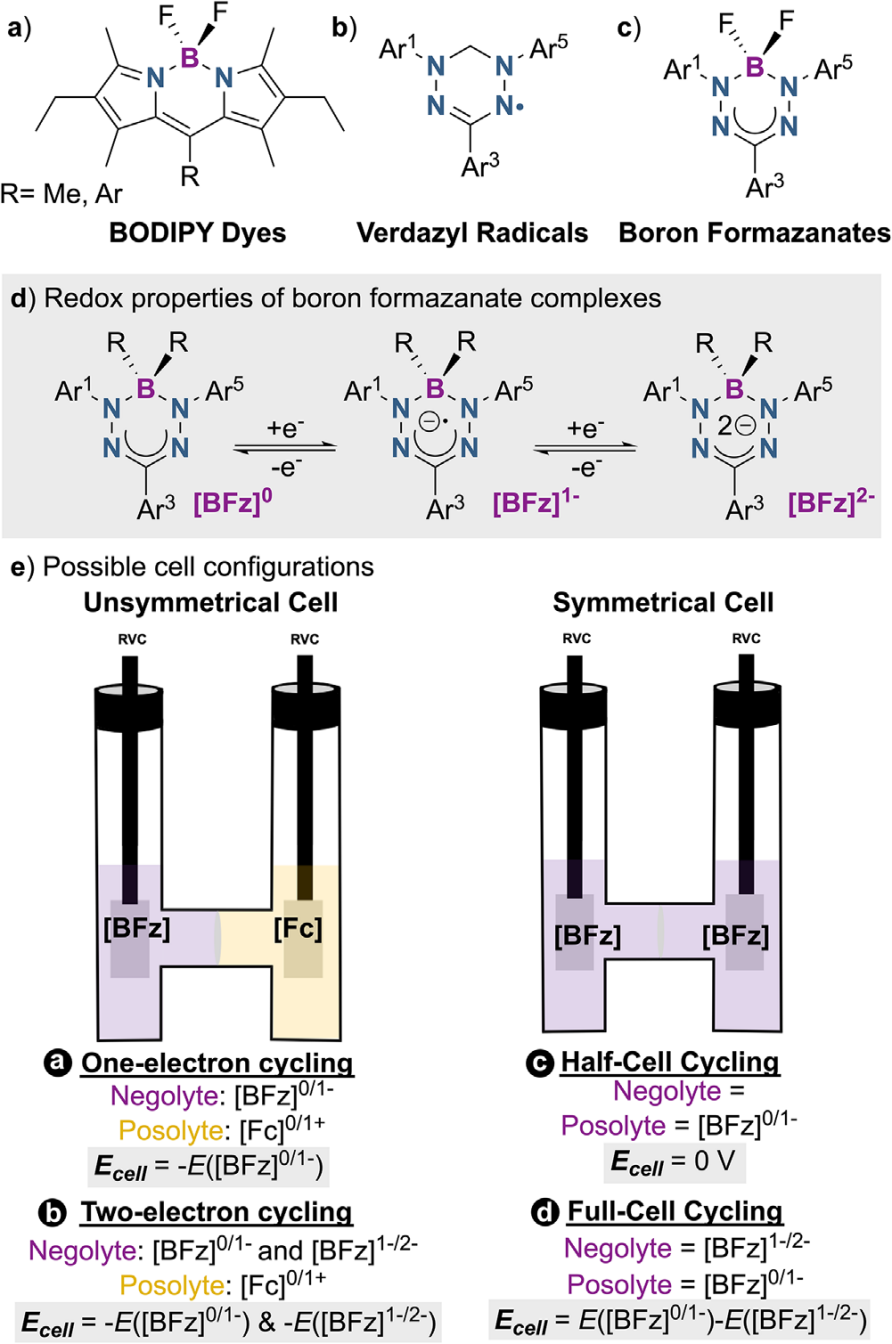
Structural comparison of (a) BODIPY dyes, (b) BF_2_‐formazanates, and (c) verdazyl radicals. (d) Neutral and reduced forms of boron‐formazanate complexes [BFz]. (e) Possible configurations for H‐cell studies. Configuration (a) unsymmetric cell with [BFz] as a one‐electron negolyte material and ferrocene (Fc) as a posolyte material. Configuration (b) unsymmetric cell with [BFz] as a two‐electron negolyte material and Fc as a posolyte material. Configuration (c) half‐cell cycling with both compartments containing an electrochemically generated equimolar mixture of [BFz]^1−^ and [BFz] (i.e. at 50% state of charge). Configuration (d) symmetric cycling with electrochemically generated [BFz]^1−^ used in both the negolyte and posolyte.

Boron‐dipyrromethene complexes (BODIPY dyes, Figure 1a) exhibit bipolar electrochemical properties that make them suitable for the construction of symmetrical batteries, with high theoretical cell voltages of >2 V [33, 34, 35]. Despite the limited stability of BODIPYs in their charged state and rapid capacity fade, this precedent nonetheless highlights the potential of boron‐based complexes in energy storage applications. With this in mind, we hypothesized that through rational ligand design, boron complexes can be systematically engineered to tune electrochemical properties and enhance overall battery cycling stability.

Boron‐formazanate complexes (Figure 1c) are an emerging class of materials known for their highly tunable optoelectronic and electrochemical properties [36]. In particular, these complexes demonstrate two reversible reduction processes, as shown by cyclic voltammetry (CV) and chemical reduction studies [37, 38]. The combination of tunability, electrochemical reversibility, and stability makes boron‐formazanate complexes promising, yet underexplored, candidates for application in NAORFBs.

Structurally related but purely organic compounds, such as verdazyl radicals (Figure 1b), were shown to have promising electrochemical performance in RFBs [39, 40]. However, their implementation is limited by poor long‐term stability, as the active radical species undergoes gradual decomposition via bimolecular hydrogen atom transfer from the heterocyclic CH_2_ position [41]. Accordingly, we wondered whether strategic replacement of this vulnerable ─CH_2_ position by a comparatively robust ─BR_2_ unit in boron‐formazanate compounds could result in improved cycling stability by introducing structural rigidity, steric protection, and inductive effects.

In this work, we systematically interrogate the electrochemical stability and battery cycling performance of a family of boron‐formazanate complexes as redox‐active materials for NAORFBs. By evaluating a series of structurally related formazanate complexes, we elucidate the factors that influence electrochemical stability. Through detailed electrochemical studies, including CV, galvanostatic cycling experiments, and postcycling degradation analysis, we identify key structural features that suppress decomposition and enhance cycling stability. Our findings provide guiding insights into the rational design of boron‐based main‐group compounds for use in NAORFBs.

## 2 Results and discussion

### 2.1 Synthesis

**FIGURE 2:**
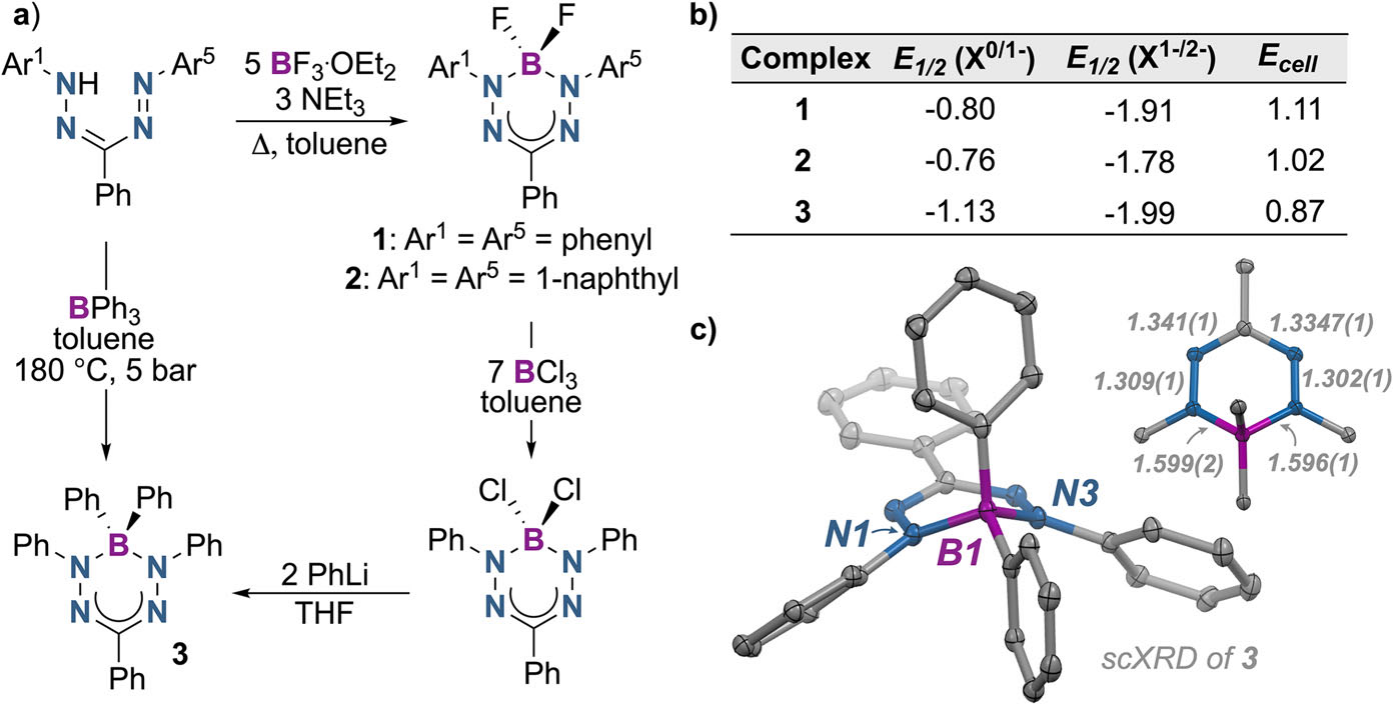
(a) Synthesis of boron‐formazanate complexes **1–3**. (b) Cyclic voltametric data (V vs. Fc^0/1+^) for 1 mM of boron‐formazanates in anhydrous MeCN, 0.1 M Bu_4_NPF_6_ supporting electrolyte. (c) Solid‐state molecular structure of boron‐formazanate **3**.

To provide a relationship between structural type and battery performance characteristics, three boron formazanates having differentiated boron and ligand substitution were considered. The synthesis of boron difluoride formazanates **1** and **2** was carried out according to literature procedures (Figure 2a) [42, 43]. The diphenylborane adduct **3** was synthesized in two ways. The first, adapting a published procedure [44] involving direct reaction of the formazan precursor with BPh_3_ at high pressure and temperature, and the second, by reacting the corresponding BCl_2_ complex with PhLi (Figure 2a). Characterization data collected for compounds **1–3** are consistent with previous reports and those collected for related compounds [42, 43, 44].

### 2.2 Electrochemical Characterization of BF2‐Formazanates

Coordination complexes with formazanates and BF_2_ derivatives in particular, exhibit ligand‐centered redox activity [36]. Consequently, charging and discharging processes necessarily involve the reversible addition or removal of electrons on the conjugated formazanate backbone. In this study, the electrochemical properties of three structurally related boron‐formazanate complexes were investigated (Figure 2a).

By cyclic voltammetry (CV; in anhydrous MeCN, 0.3 M Bu_4_NPF_6_ supporting electrolyte), the 1,3,5‐triphenyl‐substituted BF_2_‐formazanate **1** shows two reversible redox processes implicating both the formazanate radical anion and dianion (Figure 1d), with half‐wave potentials (*E*_1/2_) of ‐0.80 V for the first reduction (**1^0/1−^**) and ‐1.91 V for the second reduction (**1^1‐/2−^**) (Figure 2b; all potentials referenced vs. Fc^0/1+^). This provides a cell voltage of 1.11 V, slightly higher than the corresponding verdazyl radical (1.02 V) [42]. An analysis of the diffusion coefficient (D) and the standard rate constant (*k* ^0^) from the CV data at different scan rates (using Randles‐Ševčík and Nicholson methods [45], respectively) gave D ≈ 10^−6^ cm^2^ s^−1^; k^0^ ≈ 10^−2^ cm s^−1^ (see Supporting Information). Despite significant structural reorganization upon reduction (as indicated by X‐ray diffraction studies, and large Stokes shifts observed for these compounds) [43], the kinetics of heterogenous electron transfer is comparable to other organic redox systems and significantly faster than vanadium or other transition metal ion systems [46, 47].

We next studied a second BF_2_‐formazanate **2**, which features two N‐substituted 1‐naphthyl groups [43]. This structural modification was proposed to enhance electron delocalization, reduce spin density, and shift the reduction potentials to more positive values. Indeed, a CV of 1 mM **2** in anhydrous acetonitrile confirms this prediction (Figure 2b), showing two reversible redox events with half‐wave potentials of ‐0.76 V (**2^0/1−^**) and ‐1.78 V (**2^1‐/2−^**). The diffusion coefficient and standard rate constants were similar to **1** (D ≈ 10^−6^ cm^2^ s^−1^ and k^0^ ≈ 10^−3^ cm s^−1^) (see Supporting Information).

The final complex considered was a BPh_2_‐formazanate derivative (**3**) in which the BF_2_ group was substituted for a BPh_2_ unit, allowing for assessment of boron primary coordination sphere (e.g., B‐F vs. B‐Ph) effects on redox behavior. A CV of **3** in anhydrous acetonitrile displays two reduction waves with E_1/2_ values of ‐1.13 V (**3^0/1−^**) and ‐1.99 V (**3^1‐/2−^**) versus Fc^0/1+^. These more negative reduction potentials, relative to the BF_2_ complexes, indicate a weaker inductive effect of the phenyl groups. Analysis of CV data using different scan rates resulted in D ≈ 10^−6^ cm^2^ s^−1^; *k^0^* ≈ 10^−3^ cm s^−1^ confirming similar electrochemical kinetics across the series **1–3** (see Supporting Information).

### 2.3 Battery Cycling Performance for 1

**FIGURE 3:**
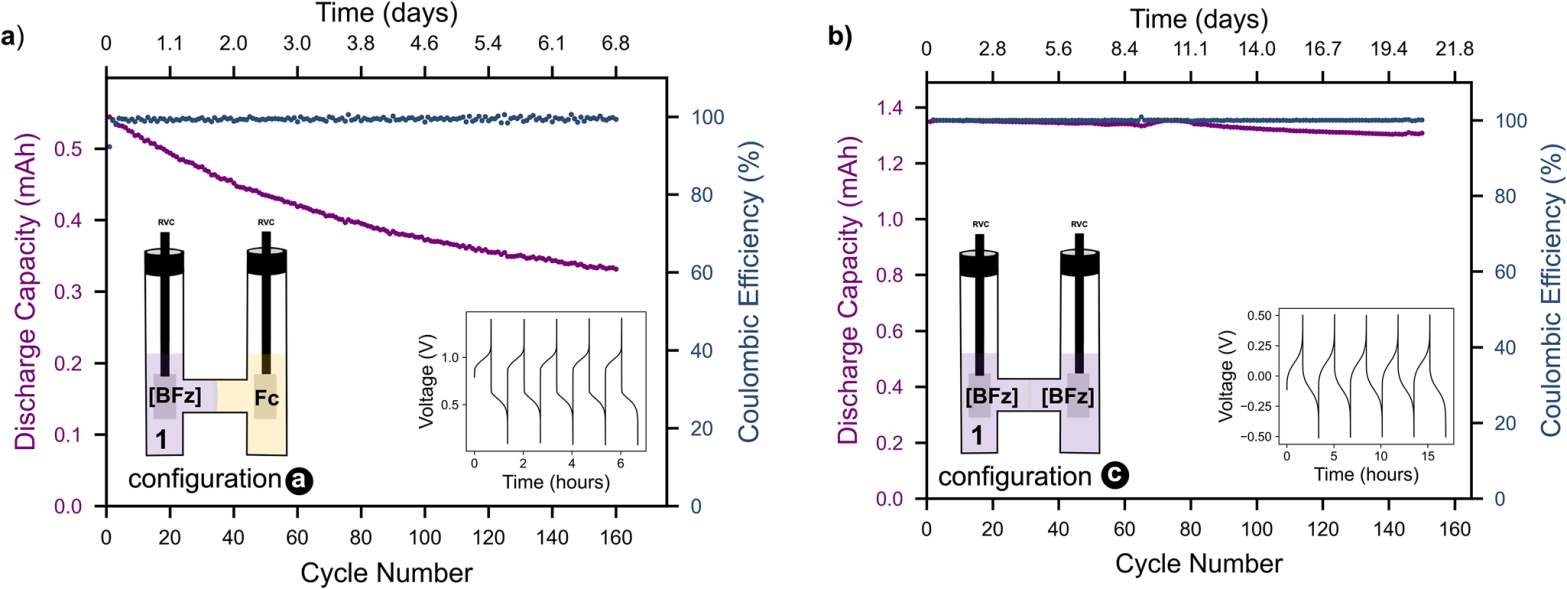
Discharge capacity and coulombic efficiency versus cycle number. (a) An asymmetrical H‐cell battery with 5 mM solution of complex **1** (Negolyte) and a 10 mM solution of ferrocene (Posolyte) in 0.3 M Bu_4_NPF_6_/MeCN (cutoff voltages of 1.4 V and 0.1 V for charging and discharging, respectively, current ±0.8 mA, theoretical capacity of 0.67 mAh. Total capacity fade of 39% after 160 cycles (b) A symmetrical H‐cell battery with 10 mM of a 50% SOC solution of **1** in 0.3 M Bu_4_NPF_6_/MeCN as both negolyte and posolyte (cutoff voltages of ±0.5 V for charging and discharging, current ±0.8 mA, theoretical capacity of 1.34 mAh. Total capacity fade of 3.25% after 150 cycles. Additional MeCN was added at cycle number 65 due to evaporation. Inset shows voltage trace of the first 5 cycles.

The electrochemical performance of complex **1** as a one‐electron negolyte material was first evaluated in a stirred H‐cell configuration with a porous frit separator. The H‐cell was assembled using 5 mL of a 5 mM solution of complex **1** as the negolyte and 5 mL of a 10 mM solution of ferrocene as the posolyte, both in anhydrous MeCN containing 0.3 M Bu_4_NPF_6_ as the supporting electrolyte. The cell was charged galvanostatically (current ±0.8 mA) to cutoff voltages of 1.4 V and 0.1 V for charging and discharging, respectively. In this cell configuration (Figure 1e configuration a), the negolyte (**1^0/1−^**) is the capacity limiting side with a theoretical capacity of 0.67 mAh, which allowed us to focus on the stability of the **1^0/1−^** redox couple in a battery context. The cell was cycled for 7 days (140 cycles) which resulted in a capacity fade of 39% (Figure 3a). To evaluate the cause of capacity loss, postcycling analysis of the electrolyte solutions was performed. The CV showed that, as expected, the porous frit separator resulted in significant material crossover, which was quantified based on CV peak currents to account for ∼38% fade. Thus, nearly all capacity loss can be attributed to active material crossover, and the chemical stability of the **1^0/1−^** couple is excellent in this cell configuration (Figure S15).

To remove crossover as a contributor to capacity fade, we performed a study of the **1** ^**0/1−**^ half‐cell in isolation using a symmetrical cycling experiment with an electrochemically generated equimolar mixture **1** ^**1−**^ and **1** (i.e. at 50% state of charge; Figure 1e configuration c). As shown in Figure 3b, cycling of the **1** ^**0/1−**^ half‐reaction in 0.3 M Bu_4_NPF_6_/ MeCN over 21 days (150 cycles) demonstrated excellent stability, with a total capacity fade of only 3% (0.14% per day). CV and NMR spectroscopy of the postcycling solutions confirmed the high stability of the **1** ^**0/1−**^ redox couple (Figures S19–21).

**FIGURE 4:**
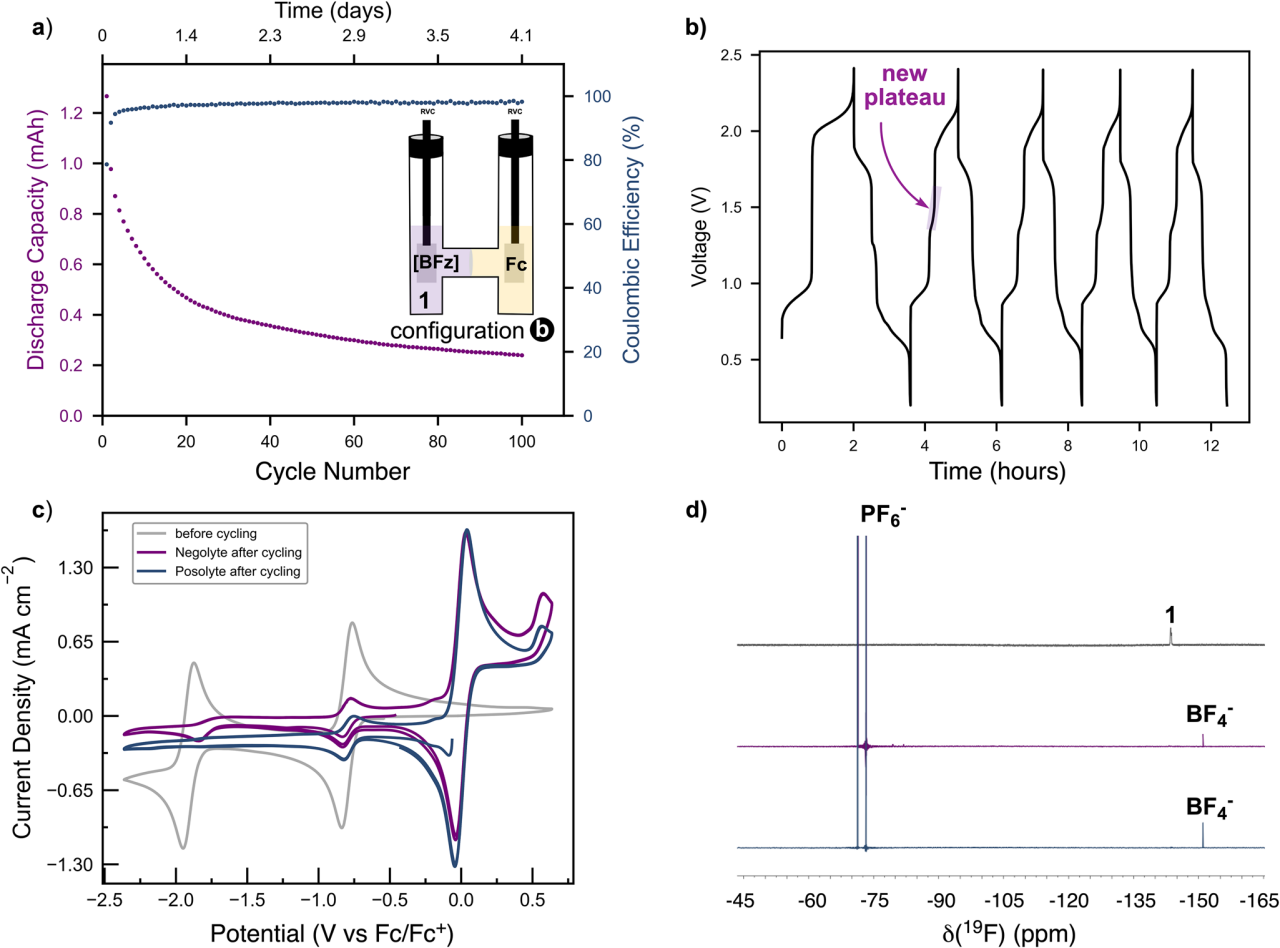
(a) Discharge capacity and coulombic efficiency versus cycle number of an asymmetrical H‐cell battery with 5 mM solution of complex **1** (Negolyte) and a 10 mM solution of ferrocene (Posolyte) in 0.3 M Bu_4_NPF_6_/MeCN (cutoff voltages of 2.4 V and 0.1 V for charging and discharging, respectively, current ±0.8 mA, theoretical capacity of 1.34 mAh). Total capacity fade of 85% after 100 cycles. (b) Charge and discharge voltage curves for the first 5 cycles. (c) Cyclic voltammogram of the negolyte solution before cycling and of the postcycling solutions. Capacity fade caused by crossover ∼20%. (d) ^19^F‐NMR spectra before battery cycling experiment in CD_3_CN (gray) and of the postcycling solutions of the negolyte (purple) and posolyte (blue).

Next, we investigated the two‐electron chemistry of complex **1**. To probe the second redox event (i.e., the **1^1‐/2−^** couple), the cutoff voltage was increased to 2.4 V during charge (Figure 1e, configuration b). In this case, a significant capacity fade of 65% due to degradation was observed after 100 cycles (Figure 4a), indicating that the dianion **1^2−^** is much less stable under these conditions.  While during the first cycle we observed two charge plateaus at the voltages expected (∼ 0.9 and 2.1 V), the subsequent discharge has an additional plateau (∼1.4 V) (Figure 4b) which indicates formation of a degradation product with a distinctly different redox potential than **1**. This newly formed discharge plateau disappeared in subsequent cycles, indicating continued decomposition. To further investigate the degradation process, the negolyte solution was analyzed postcycling. CV of the **1**‐based postcycling solution revealed irreversible behavior for the second reduction wave (Figure 4c). Furthermore, ^1^H‐NMR spectroscopic analysis revealed the appearance of new aromatic peaks, consistent with the formation of degradation products derived from the formazanate ligand (Figure S24). This is further supported by ^19^F‐NMR spectroscopy, which revealed the presence of BF_4_^−^, indicative of fluoride transfer between B centers, and loss of the organic ligand. We hypothesize that this decomposition pathway is initiated by fluoride elimination from the doubly reduced state in **1^2−^**, a pathway that has been reported previously [38].

### 2.4 Using Ligand Design Concepts for Improved Cycling Stability

To improve the stability of the dianionic boron‐formazanate species during battery cycling, structural modification of the redox‐active ligand was proposed. Specifically, increasing the π‐conjugation of the ligand was expected to promote charge delocalization, reduce spin density, and shift potentials to more positive values [43]. This was predicted to enhance stability of the dianionic form and thereby suppress decomposition pathways observed during battery cycling. Therefore, the electrochemical performance of BF_2_‐formazanate **2**, featuring 1‐naphtyl substituents at the 1‐ and 5‐positions, was evaluated. First, the stability of the one‐electron redox process of **2** versus a Fc posolyte was confirmed, using the same methodology as for complex **1** (cell configuration a; data shown in Figures S26–29). Subsequently, two electron cycling of **2** against Fc was investigated (cell configuration b). Although complex **2** showed improved performance relative to complex **1**, it maintained a significant capacity fade of 33% due to degradation after 75 cycles (5 days) (Figure S30). Postcycling analysis confirmed that the formation of BF_4_^−^ is involved in degradation. We hypothesize that dianionic BF_2_‐formazanate complexes are inherently prone to fluoride elimination, contributing to the observed instability (Figures S31–33).

To mitigate this decomposition pathway, a fluoride‐free boron formazanate complex (**3**) having a BPh_2_ unit was synthesized. Phenyl substituents are less susceptible to elimination, and their increased steric bulk (c.f., a halide) may provide additional protection to the boron center (see Figure 2c for crystal structure) [48]. Our group has previously shown that dianionic BPh_2_‐formazanate compounds exhibit enhanced stability and can be isolated [49], though the redox potentials are pushed to even more negative values (∼ ‐2 V). For complex **3**, the cycling stability of the **3^0/1−^** couple is excellent, with virtually all capacity fade attributable to crossover (Figures S34–36). A cell where two‐electron cycling versus Fc was carried out (configuration b) showed only 16% capacity fade due to degradation after 75 cycles (5 days) (Figures S37,38), confirming that replacement of the susceptible fluoride groups with phenyl substituents reduces the rate of decomposition.

### 2.5 Performance of 3 as a Bipolar Material

Given the promising performance of **3** as an anolyte, we subsequently investigated its potential as a bipolar redox‐active battery material. The radical anion **3** ^**1−**^ material can be reversibly reduced to **3^2−^** or oxidized to **3^0^**, which makes it suitable for application in symmetrical batteries, that is, with identical electrolyte composition on the positive and negative side. In these symmetrical batteries (Figure 1e, configuration d), capacity fade due to active material crossover is mitigated and lifetime can be significantly improved by polarity inversion to ‘reset’ active material imbalance [50, 51]. For this experiment, a 5 mM solution of **3^1−^** was electrochemically generated and evenly distributed between the two compartments of an H‐cell and cycled continuously (cutoff voltages of 1.4 V and 0.1 V for charging and discharging, respectively; current ±0.8 mA; theoretical capacity 0.67 mAh). After 100 cycles (4.5 days), a capacity retention of 60% was observed (Figure S40). Based on CV of the postcycling solutions, we identified that the redox couple **3**/**3^1−^** is more stable than the corresponding **3^1−^**/**3^2−^** redox couple (Figure S41). This is consistent with the data obtained from asymmetric H‐cell cycling, where two‐electron cycling of **3** versus a Fc posolyte showed decreased stability (*vide supra*).

### 2.6 Battery Cycling in a Flow Setup

**FIGURE 5:**
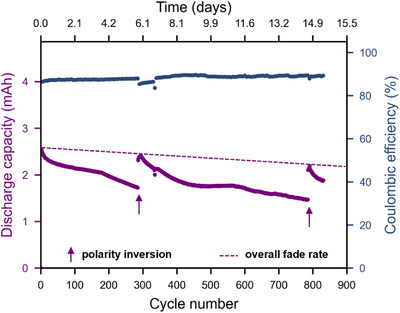
Discharge capacity and efficiency versus cycle number of a flow cell battery with 20 mM of **3^1−^** in 0.3 M Bu_4_NPF_6_/ MeCN as both negolyte and posolyte with a polarity inversion at cycle 285 and 785 (cutoff voltages of 1.4 and 0.1 V for charging and discharging respectively, current density ±3.9 mA/cm^2^, theoretical capacity of 3.2 mAh).

Based on the H‐cell cycling data described above, complex **3** was selected for testing in a flow cell setup. Symmetrical flow cell cycling was carried out using electrochemically generated solutions of **3** ^**1−**^ (20 mM in 0.3 M Bu_4_NPF_6_/ MeCN). The cell was galvanostatically charged (3.9 mA/cm^2^) with voltaic cutoffs at 1.4 and 0.1 V. Under these conditions, the cell was cycled 285 times (5.7 days) with a capacity retention of 66% (Figure 5).

To highlight the intrinsic self‐balancing nature of symmetric redox flow systems, polarity inversion was applied after this initial period, which resulted in a restoration to 90% of the original capacity. The flow cell was continued for an additional 500 cycles and yielded 57% capacity retention over a total of 14.5 days of charge‐discharge cycling (Figure 5). A second polarity inversion subsequently achieved a discharge capacity that is 85% of the initial value, based on which we estimate a fade rate of ca. 1% per day (0.018% per cycle). Reversal of battery polarity thus results in a substantial boost in capacity, demonstrating its beneficial effect for the lifetime of symmetrical batteries.

The superior cycling performance of **3^1−^** in a flow cell setup compared to the standard H‐cell is likely attributed to differences in the cell architecture and the resulting levels of contamination. In an H‐cell, the two compartments are separated by a porous glass frit, which might accumulate trace impurities across multiple experiments and may result in undesirable reactions. In contrast, in flow cells a fresh membrane (Daramic in this case) is used for each experiment, minimizing the risk of adventitious residues that negatively affect battery performance.

Contextualizing these results alongside verdazyl and BODIPY systems allows for a clear assessment of the performance. Relative to BODIPY dyes, which typically lose nearly all capacity after ∼40 cycles, compound **3^1−^** exhibits improved stability even though the redox potential of the negolyte reaction (**3^1‐/2−^**) is over 300 mV more negative than related BODIPYs [33]. A comparison to the parent bipolar verdazyl radicals shows that **3** exhibits improved cycling stability. Thus, substitution of the labile CH_2_ group in verdazyls, which is prone to C─H bond homolysis [41], with the BPh_2_ unit in **3** leads to enhanced battery lifetime, especially when incorporating polarity inversion in the cycling protocol.

## 3 Conclusion

This work introduces boron‐formazanates as a chemically tunable and electrochemically robust class of redox‐active materials for nonaqueous organic redox flow batteries (NAORFBs). Comprehensive electrochemical profiling, coupled with postmortem analysis, uncovers B─F bond cleavage in the two‐electron reduced state as a key degradation pathway contributing to capacity fade. By leveraging rational design through steric and electronic modulation, a diphenylboron derivative was developed that exhibits markedly enhanced cycling stability. Notably, flow cell experiments reveal reversible capacity recovery via polarity inversion in a symmetric cell architecture, demonstrating an operationally simple electrolyte rebalancing strategy for bipolar materials. Together, these results establish boron‐formazanates as a versatile redox platform and provide critical design principles for advancing boron‐based active materials in NAORFBs.

## Conflicts of Interest

The authors declare no conflict of interest.

## Supporting information



Experimental procedures and spectroscopic data for all complexes. The authors have cited additional references within the Supporting Information [52–59].

## Data Availability

The data that support the findings of this study are available from the corresponding author upon reasonable request.
