# Supplementary material for: Boron‐Formazanate Complexes as Tunable Redox‐Active Materials for Non‐Aqueous Redox Flow Batteries
Source: Chemistry. 2026 Jan 5;32(9):e03592. doi: 10.1002/chem.202503592 (PMC12958094; doi:10.1002/chem.202503592)
Supplement: Supplementary file 1 — Experimental procedures and spectroscopic data for all complexes. The authors have cited additional references within the Supporting Information [52–59]. [file CHEM-32-e03592-s001.docx]

**Supporting information for**

**Boron-Formazanate Complexes as Tunable Redox-Active Materials for Non-Aqueous Redox Flow Batteries**

Reinder H. Bouma,^[a]^ Mitchell J. Demchuk,^[b]^ Suhjung Chun,^[b]^ Francis L. Buguis,^[b]^ Erin L. Cotterill,^[b]^ Arvin M. Mehdian,^[b]^ Paul D. Boyle,^[b]^ Marcus W. Drover,*^[b]^ Joe B. Gilroy,*^[b]^ and Edwin Otten*^[a]^

**Contents**

[Materials and methods 2](#_heading=h.f7tuyv9sge3y)

[General considerations 2](#_heading=h.n6owit5b3g3j)

[Electrochemical experiments 3](#_heading=h.tnx5zntmsj4j)

[Experimental 4](#_heading=h.cj6g2ngcsst9)

[NMR spectroscopy 5](#_heading=h.ywgopuu5k0ls)

[UV-Vis Spectroscopy 8](#_heading=h.ln18v8i9t1vl)

[FT-IR Spectroscopy 9](#_heading=h.8svi95y8d10u)

[Cyclic voltammetry 10](#_heading=h.q8840sjhpnv5)

[H-cell battery test with post-cycling analysis 12](#_heading=h.gdjedsqmjpjv)

[Redox flow battery testing 28](#_heading=h.5f8xew6xqqtw)

[Determination of diffusion coefficients and standard rate constants by cyclic voltammetry 29](#_heading=h.4q9djaglqmq8)

[Single Crystal Structure Analysis 33](#_heading=h.8j6dbzgxuyt2)

[References 35](#_heading=h.g1d2gvlk6629)

# Materials and methods

## General considerations

Reactions were performed under an N_2_ atmosphere using standard Schlenk techniques unless otherwise stated. Reagents were purchased from Sigma-Aldrich, Alfa Aesar, Strem Catalog or Oakwood Chemicals Inc. and used as received. Solvents were purchased from Caledon Laboratories, dried using an Innovative Technologies Inc. solvent purification system, collected under vacuum, and stored under N_2_ over 3 Å molecular sieves. An Anton Paar Monowave 50 was used for carrying out solvothermal reactions. The synthesis of boron difluoride formazanate complexes **1** and **2** as well as their formazan precursors have been reported previously.^[52,53]^ The supporting salt Bu_4_NPF_6_ (≥99.0%, for electrochemical analysis) used for the cyclic voltammetry studies and battery cycling experiments was obtained from Sigma-Aldrich and stored under N_2_ atmosphere in a glovebox. Acetonitrile (Thermo Scientific Chemicals, Extra Dry over Molecular Sieve, 99.9%) was stored under N_2_ atmosphere in a glovebox.

NMR spectra were recorded on 400 MHz (^1^H: 399.8 MHz, ^11^B: 128.3 MHz, ^19^F: 376.1 MHz) Bruker AVIII HD 400 and Varian Mercury Plus 400 spectrometers. ^1^H NMR spectra were referenced to residual tetramethylsilane (δ: 0.00) using the residual CDCl_3_ (δ: 7.26), DMSO-*d_5_* (δ: 2.50) or CD_3_CN solvent signals and ^13^C{^1^H} NMR spectra were referenced to tetramethylsilane (δ:0.0) using the CDCl_3_ (δ: 77.0) solvent signal. ^11^B NMR spectra were referenced to BF_3_·OEt_2_ (δ: 0.0). ^19^F spectra were referenced to CFCl_3_ (δ: 0.0). Mass spectrometry data were recorded in positive-ion mode using a high-resolution Orbitrap Exploris 120 mass spectrometer using atmospheric pressure chemical ionization or liquid injection field desorption ionization. FT-IR spectra were recorded on a PerkinElmer Spectrum Two instrument using an attenuated total reflectance accessory. Solution UV-vis absorption spectra were recorded using a Cary 5000 UV-Vis-NIR spectrophotometer. Molar extinction coefficients were determined from the slope of a plot of absorbance against concentration using four solutions with different known concentrations.

All electrochemical measurements were performed at ambient temperature in a glovebox under an inert N_2_ atmosphere. Cyclic voltammetry (CV) measurements were conducted using a CH Instruments Electrochemical Analyzer potentiostat (CHI600C) and data were processed with CHI600c software, applying iR compensation. H-cell cycling was performed in a custom glass H-cell using a Neware BTS4000 cycler (CT-4008T-5V 50mA-164) and data was recorded with Neware BTS software (8.0.0). Flow battery cycling was performed with a VSP-300 potentiostat (Bio-Logic) and data was recorded with EC-LAB software (V11.31).

Crossover contributions to capacity fade were quantified using Equation 1 (E1), where the ratio was taken between the anodic peak current (i_pa_) originating from the first redox wave of boron-formazanate species prior to cycling (in the negolyte) and the peak current of the same species detected in the posolyte after cycling, arising from crossover. Peak currents were determined by cyclic voltammetry.

$Crossover=\frac{i_{pa of E_{1/2}^{red1} after cycling in posolyte}}{i_{pa of E_{1/2}^{red1} before cycling in negolyte}}$ *(E1)*

## Electrochemical experiments

**Cyclic voltammetry**

Cyclic voltammetry was performed in a three-electrode configuration consisting of a Pt wire counter electrode, a leakless Ag/AgCl reference electrode (EDAQ, ET072) and a GC working electrode (diameter = 3 mm). The GC working electrode was polished before the experiment using an alumina slurry (0.03 μm), rinsed with distilled water and subjected to brief ultrasonication to remove any adhered alumina microparticles. The CV data was referenced to ferrocene in acetonitrile.

**Charge/discharge cycling in H-cells**

Charge–discharge tests were performed in a custom H-cell with high surface area to volume ratio. The cell consisted of two electrolyte chambers separated by a P5 glass frit (area of ∼1.6 cm^2^) to minimize crossover. Reticulated vitreous carbon (Duocel, 45 ppi) was used as electrodes with an interelectrode distance of about 20 mm. For the battery tests, the electrolyte chambers were loaded with 5 mL of 5 mM active species in 0.3 M supporting electrolyte salt and stirred continuously. An Ohmic resistance of about 200 to 300 Ω was measured in all tests.

**Charge/discharge cycling using a single half-reaction in a H-cell**

In a N_2_-filled glovebox, one side of a glass H-cell was filled with 5 mL of a 10 mM solution of complex **1** in acetonitrile containing 0.3 M supporting electrolyte salt. The other side of the glass H-cell was filled with 5 mL of a 20 mM ferrocene solution. Charging was carried out using a constant current of 0.8 mA, until the voltage reached 1.3 V. After that, charging was continued at a constant voltage of 1.3 V, until the current had dropped below 0.1 mA. The electrochemically generated solution of 10 mM **1^-^** was mixed with a solution of **1** containing the same supporting electrolyte salt to make up an equimolar mixture of neutral and charged state in 0.3 M supporting salt. The 50% SOC mixture that is thus obtained was equally distributed over the two sides of a new H-cell, and charge-discharge cycling was carried out in constant current mode with cut-off voltages of +0.5 V and -0.5 V.

**Charge/discharge cycling experiment using a symmetrical composition**

In a N_2_-filled glovebox, one side of a glass H-cell was filled with 5 mL of a 10 mM solution of active species in acetonitrile containing 0.3 M supporting electrolyte salt. The other side of the glass H-cell was filled with 5 mL of a 12 mM ferrocene solution. Charging was carried out using a constant current of 0.8 mA, until the cutoff voltage was reached. After that, charging was continued at the cutoff voltage, until the current had dropped below 0.1 mA. The electrochemically generated solution of reduced active material was diluted with 5 mL of acetonitrile containing 0.3 M supporting electrolyte salt. This solution was equally distributed over the two sides of a new H-cell, and charge-discharge cycling was carried out in constant current mode with continuous stirring.

**Flow cell test**

In a N_2_-filled glovebox, one side of a glass H-cell was filled with 12 mL of a 20 mM solution of active species in acetonitrile containing 0.3 M supporting electrolyte salt. The other side of the glass H-cell was filled with 12 mL of a 22 mM ferrocene solution. Charging was carried out using a constant voltage 1.5 V, until the cutoff current dropped below 0.2 mA. The flow cell measurement was carried out using a zero-gap flow cell.^[54]^ The flow battery was assembled outside the glovebox. A combination of a graphite charge-collecting plate and three layers of a nonwoven carbon paper electrode with an area of 2.55 cm^2^ (Sigracet 29AA) was put on either side of the flow cell. The cell was sealed using two Gore-tex ePTFE gaskets (0.5 mm). The two half cells were separated by a Daramic 175 porous membrane. The gasket window provided for a 2.55 cm^2^ exposed area of the membrane which was used as the active area of the flow cell. The cell was connected to a peristaltic pump (LeadFluid) by Masterflex norprene pump tubing (Masterflex™ 06404-14), and the electrochemically generated electrolyte solution (6 mL of 20 mM of **3^1-^** in 0.3 M NBu_4_/PF_6_ in acetonitrile on both sides) were pumped through the cell using a flow rate of 15 mL/min).

# Experimental

**Synthesis of boron dichloride 1,3,5-triphenylformazanate complex**

To a solution of boron difluoride formazanate **1** (0.350 g, 1.01 mmol) in toluene (30 mL), boron trichloride (1 M in heptane, 7.04 mL, 7.04 mmol) was added dropwise. The solution was stirred for 6 h before water (15 mL) was added to quench any unreacted boron trichloride. Then, the mixture was transferred to a separatory funnel, and the organic layer was washed with water (3 × 100 mL). The organic layer was dried over MgSO_4_, gravity filtered, and the solvent was removed *in vacuo*. The residue was washed with ice-cold pentane, and the boron dichloride formazanate adduct was isolated as a red solid using vacuum filtration. Yield = 0.148 g, 39%. M.P.:138–140 °C. ^1^H NMR (400 MHz, C_6_D_6_) δ 8.00–7.98 (m, 2H), 7.83–7.81 (m, 4H), 7.14 (d, ^3^*J*_HH_ = 2 Hz, 2H), 7.00–6.97 (m, 7H). ^11^B NMR (128 MHz, C_6_D_6_) δ 2.90. ^13^C{^1^H} NMR (101 MHz, C_6_D_6_) δ 151.8, 145.2, 133.2, 130.1, 129.9, 129.1, 128.6, 126.2, 125.8. FT-IR (ATR): 1487 (w), 1344 (w), 1282 (w), 1259 (w), 1212 (m), 1157 (w), 1114 (w), 976 (w), 924 (m), 922 (m), 845 (w), 765 (s), 731 (m), 687 (s), 664 (m), 608 (s), 530 (s) cm^–1^. UV-Vis (CH_2_Cl_2_): λ_max_ = 514 nm (ε = 13000 M^–1^ cm^–1^). Mass spec. (LIFDI, +ve mode): exact mass calculated for [C_19_H_15_BCl_2_N_4_]^+^, [M]^+^ = 380.0767; exact mass found: 380.0737; difference = –7.9 ppm.

**Synthesis of diphenylboron formazanate complex 3 using triphenyl borane**

To a solution of 1,3,5-triphenylformazan (0.060 g, 0.20 mmol) in toluene (3 mL), triphenylborane (0.097 g, 0.40 mmol) was added. The solution was heated at 190 °C at 5 bar for 30 min in a solvothermal reactor, during which time the red solution turned pink. The solvent was then removed *in vacuo*, and the resulting solids were purified using column chromatography (neutral alumina, hexane:CH_2_Cl_2_ = 9:1, R_f_ = 0.14) to yield the product as a dark red solid. Yield = 0.016 g, 17%. M.P.: 168–170 °C. ^1^H NMR (400 MHz, C_6_D_6_) δ 8.07 (d, ^3^*J*_HH_ = 7.1 Hz, 2H), 7.36–7.31 (m, 8H), 7.11 (t, ^3^*J*_HH_ = 7.4 Hz, 2H), 7.05–7.03 (m, 7H) 6.84–6.78 (m, 6H). ^11^B NMR (128 MHz, C_6_D_6_) δ 1.35 (s). ^13^C NMR (101 MHz, C_6_D_6_) δ 154.0, 146.9, 143.7, 135.2, 134.2, 129.2, 128.8, 128.5, 128.3, 127.6, 127.4, 126.8, 125.6. FT-IR (ATR): 3069 (w), 3044 (w), 3006 (w), 1588 (w), 1486 (w), 1432 (w), 1347 (m), 1281 (m), 1242 (s), 1210 (m), 1147 (m), 1106 (w), 1070 (m), 1000 (w), 940 (m), 883 (m), 759 (s), 743 (m), 685 (s), 656 (m) cm^–1^. UV-vis (CH_2_Cl_2_): λ_max_ = 503 nm (ε = 14400 M^–1^ cm^–1^). Mass spec. (APCI, +ve mode): exact mass calculated for [C_31_H_26_BN_4_]^+^, [M+H]^+^ = 465.2251; exact mass found: 465.2241; difference = –2.1 ppm.

**Synthesis of diphenyl boron formazanate 3 using phenyllithium**

To a solution of boron dichloride 1,3,5-triphenylformazanate complex (0.200 g, 0.525 mmol) in THF (30 mL), phenyl lithium (1.9 M in dibutyl ether, 0.550 mL, 1.05 mmol) was added dropwise. The solution was stirred for 3 h. The reaction mixture was then filtered through Celite, and the filtrate was concentrated *in vacuo*. The crude solid was purified using column chromatography (neutral alumina, hexane:CH_2_Cl_2_ = 5:1, R_f_ = 0.43). The solvent was removed *in vacuo* to yield the product as a dark red solid. Yield = 0.028 g, 12%. The characterization data collected were consistent with those reported above.

# NMR spectroscopy


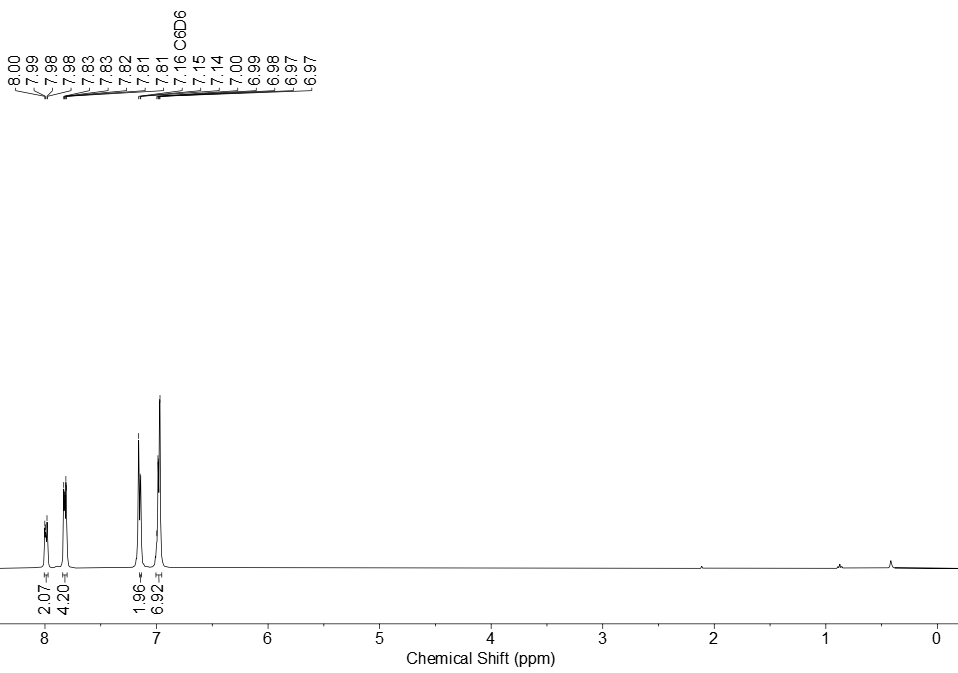

**Figure S1**. ^1^H NMR spectrum of BCl_2_ formazanate complex recorded in C_6_D_6_.


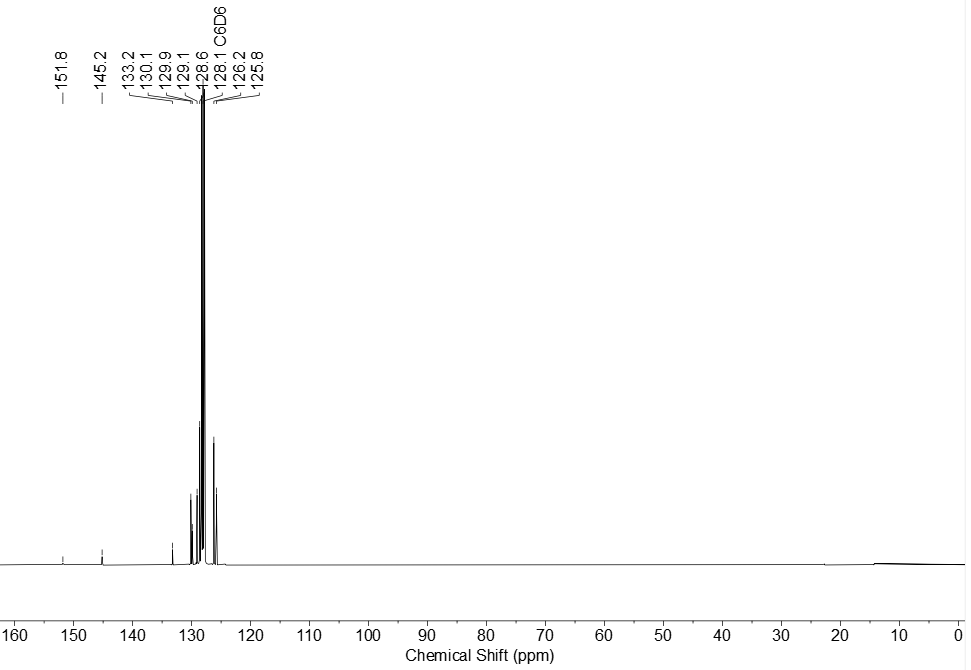

**Figure S2**. ^13^C{^1^H} NMR spectrum of BCl_2_ formazanate complex recorded in C_6_D_6_.


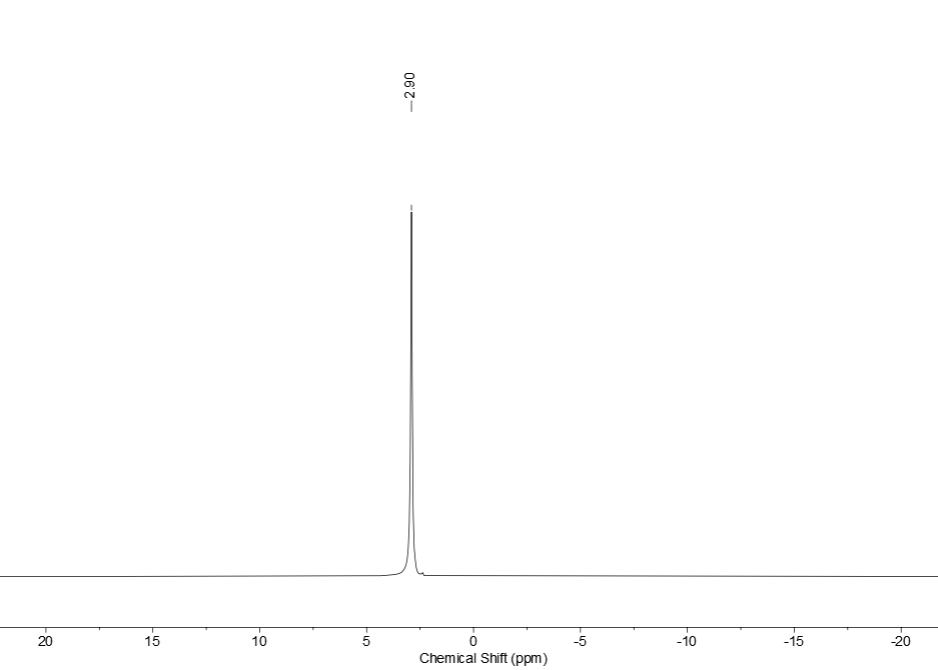

**Figure S3**. ^11^B NMR spectrum of BCl_2_ formazanate complex recorded in C_6_D_6_.


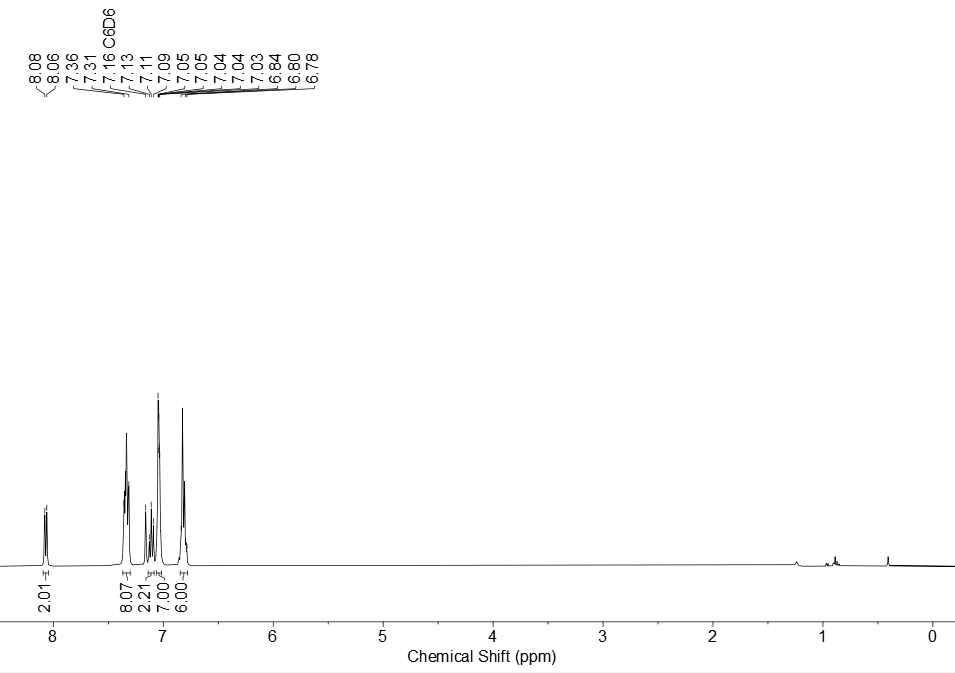

**Figure S4**. ^1^H NMR spectrum of diphenyl boron formazanate complex **3** recorded in C_6_D_6_.


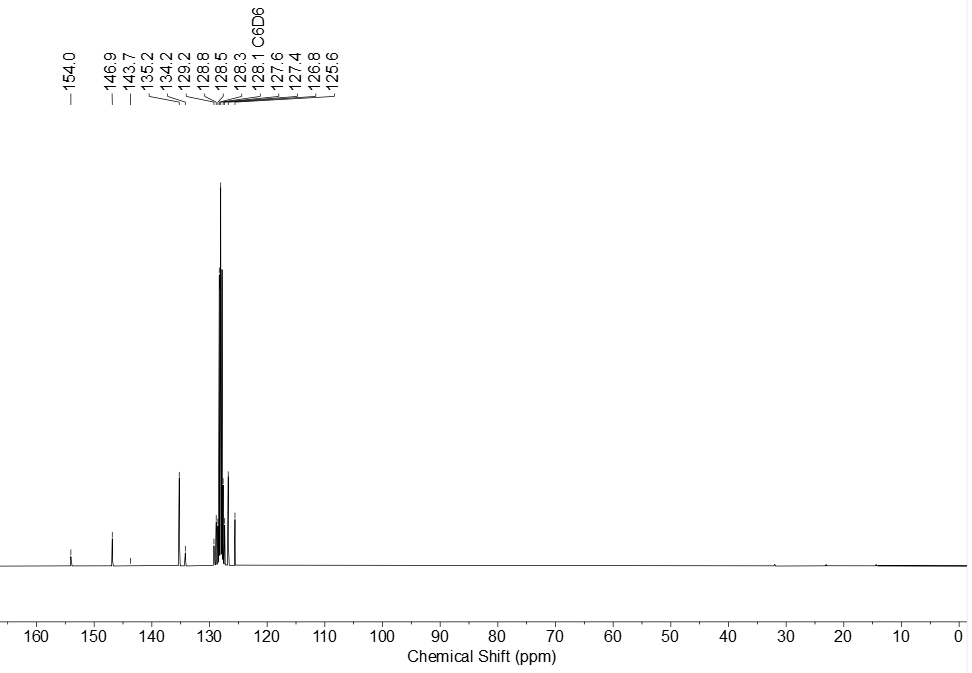

**Figure S5**. ^13^C{^1^H} NMR of diphenyl boron formazanate complex **3** recorded in C_6_D_6_.


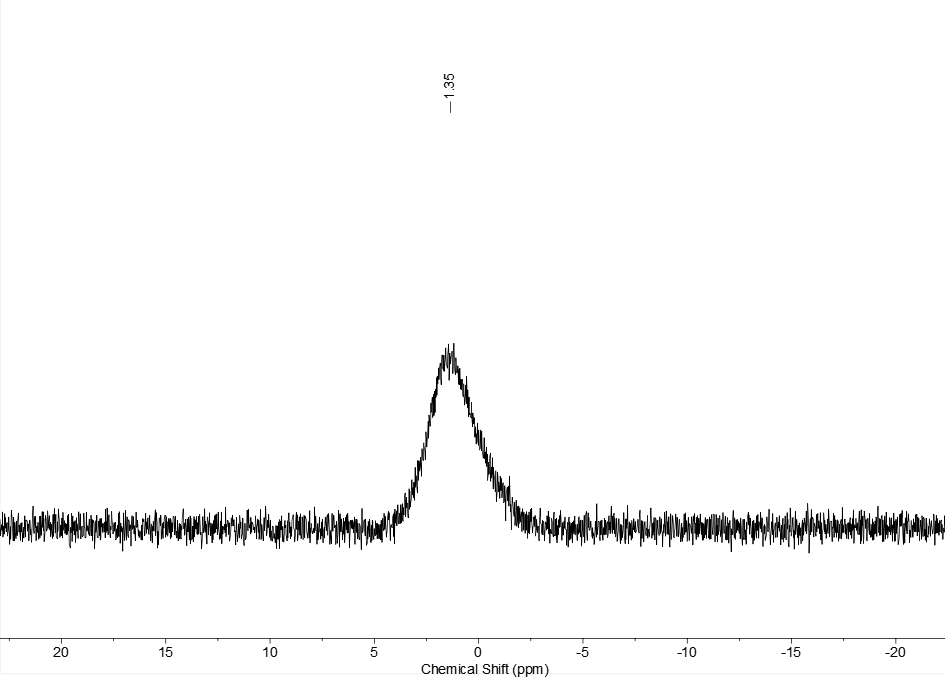

**Figure S6**. ^11^B NMR spectrum of diphenyl boron formazanate complex **3** recorded in C_6_D_6_.

# UV-Vis Spectroscopy

**Figure S7**. UV-Vis absorption spectrum of BCl_2_ formazanate complex in CH_2_Cl_2_.

**Figure S8**. UV-Vis absorption spectrum of diphenyl boron formazanate complex **3** in CH_2_Cl_2_.

# FT-IR Spectroscopy

**Figure S9**. FT-IR spectrum of BCl_2_ formazanate complex.

**Figure S10**. FT-IR spectrum of diphenyl boron formazanate complex **3**.

# Cyclic voltammetry


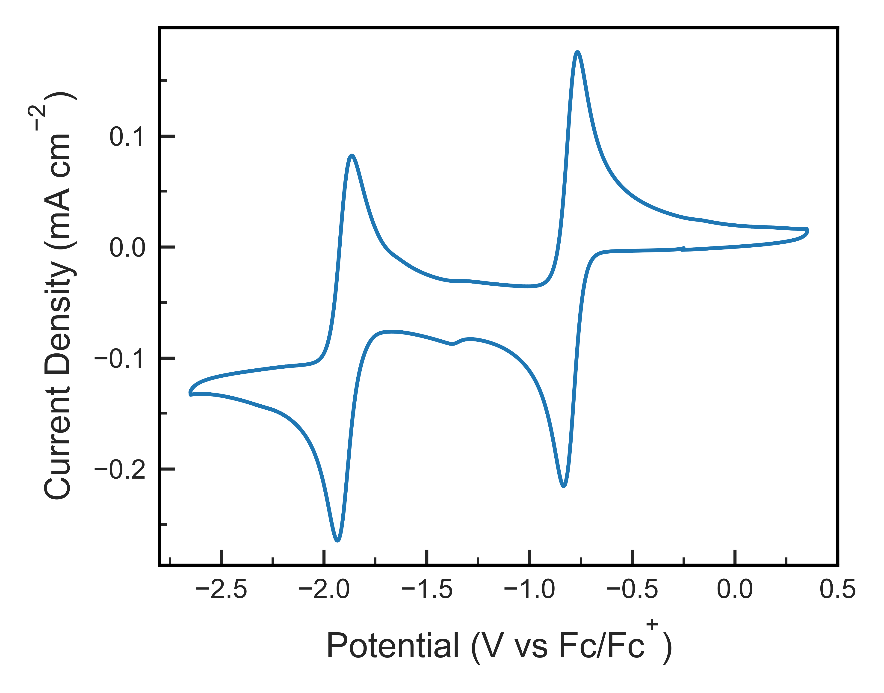


**Figure S11**. Cyclic voltammogram of 1 mM complex **1** recorded at 50 mV s^-1^ in MeCN with 0.1 M Bu_4_NPF_6_ as the supporting electrolyte.

**
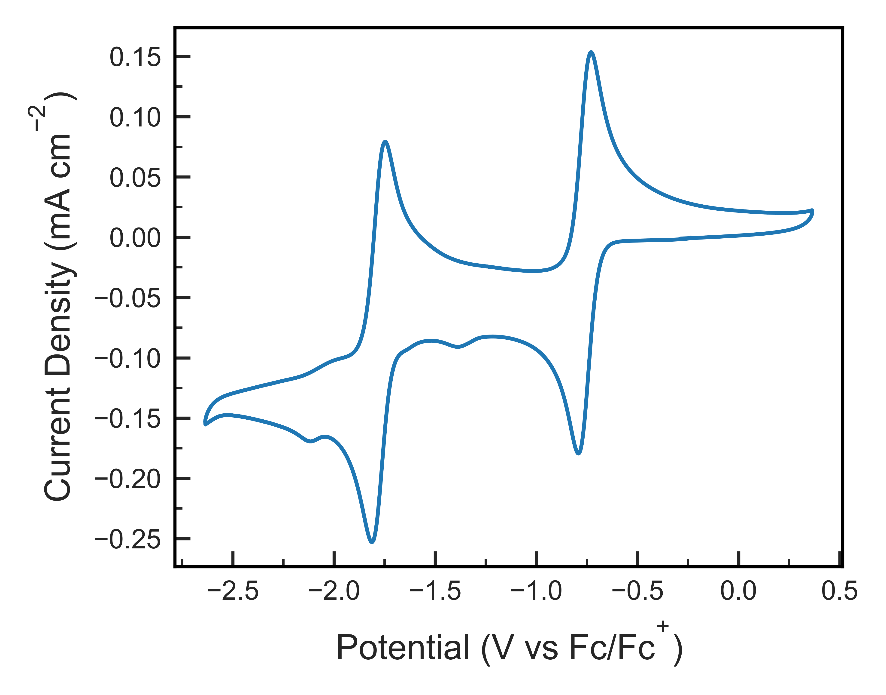
**

**Figure S12**. Cyclic voltammogram of 1 mM complex **2** recorded at 50 mV s^-1^ in MeCN with 0.1 M Bu_4_NPF_6_ as the supporting electrolyte.

**
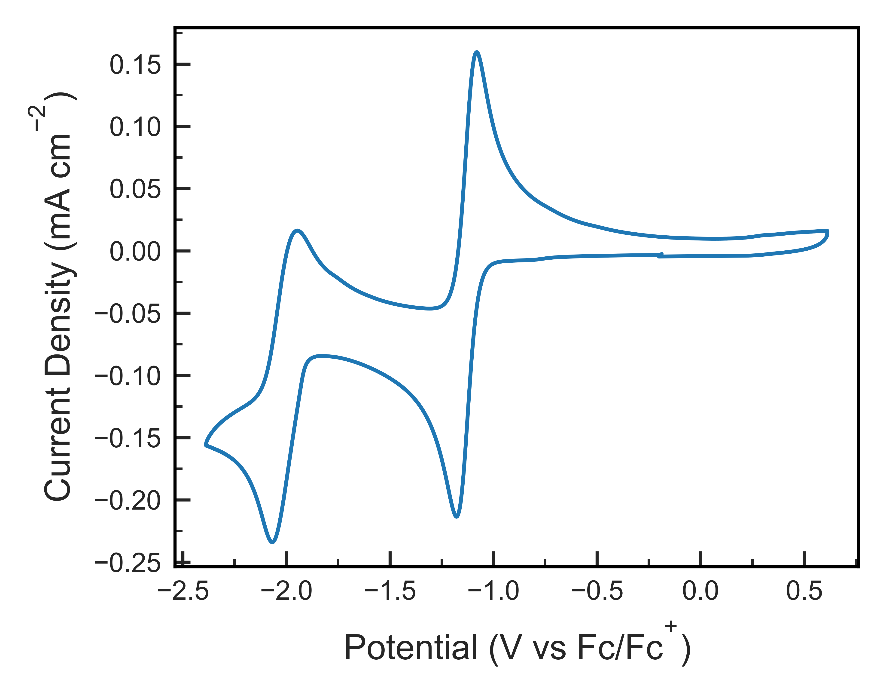
**

**Figure S13**. Cyclic voltammogram of 1 mM complex **3** recorded at 50 mV s^-1^ in MeCN with 0.1 M Bu_4_NPF_6_ as the supporting electrolyte.

# H-cell battery test with post-cycling analysis



**Figure S14.** (left) Charge and discharge voltage curves for the first 5 cycles of a H-cell battery with 5 mM of **1** (negolyte) and 10 mM of Fc (posolyte) in 0.3 M [Bu_4_N][PF_6_]/MeCN (cutoff voltages of 1.4 V and 0.1 V for charging and discharging, respectively, current ±0.8 mA). (right) discharging capacities (theoretical capacity of 0.67 mAh) and coulombic efficiency for each cycle. Total capacity fade of 39% after 160 cycles.


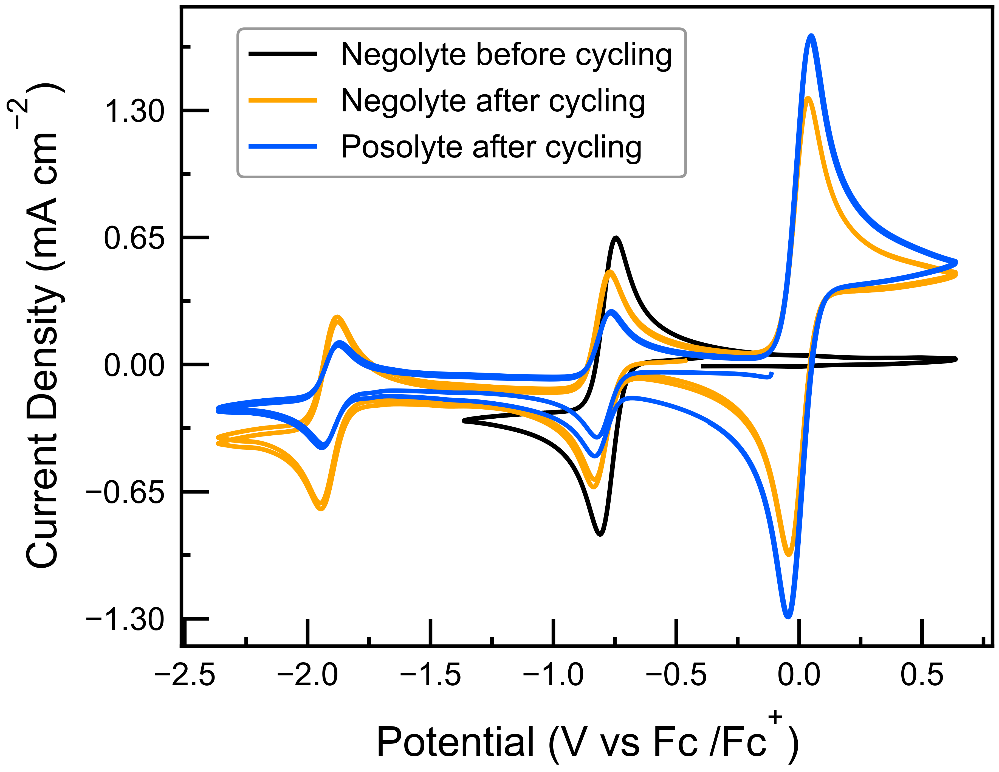


**Figure S15.** Cyclic voltammogram of the negolyte solution before one-electron cycling with **1** (negolyte) and 10 mM of Fc (posolyte) in 0.3 M [Bu_4_N][PF_6_]/MeCN and of the post-one-electron-cycling solutions. From the peak currents obtained from cyclic voltammetry, capacity fade caused by crossover was estimated to be 38%.

**Figure S16.**  ^1^H-NMR spectra of **1** in CD_3_CN (black) and of the post-one-electron-cycling of the negolyte (orange) and posolyte (blue) of a battery with a 5 mM solution of complex **1** (Negolyte) and a 10 mM solution of ferrocene (Posolyte) in 0.3 M [Bu_4_N][PF_6_]/MeCN.

**Figure S17.**  ^19^F-NMR spectra of **1** in CD_3_CN (black) and of the post-one-electron-cycling of the negolyte (orange) and posolyte (blue) of a battery with a 5 mM solution of complex **1** (Negolyte) and a 10 mM solution of ferrocene (Posolyte) in 0.3 M [Bu_4_N][PF_6_]/MeCN.



**Figure S18.** (left) Charge and discharge voltage curves for the first 5 cycles of a symmetrical H-cell battery with 10 mM of a 50% SOC solution of **1** in 0.3 M [Bu_4_N][PF_6_]/MeCN (cutoff voltages of ±0.5 V for charging and discharging, current ±0.8 mA). (right) discharging capacities (theoretical capacity of 1.34 mAh) and coulombic efficiency for each cycle. Total capacity fade of 3% after 150 cycles.


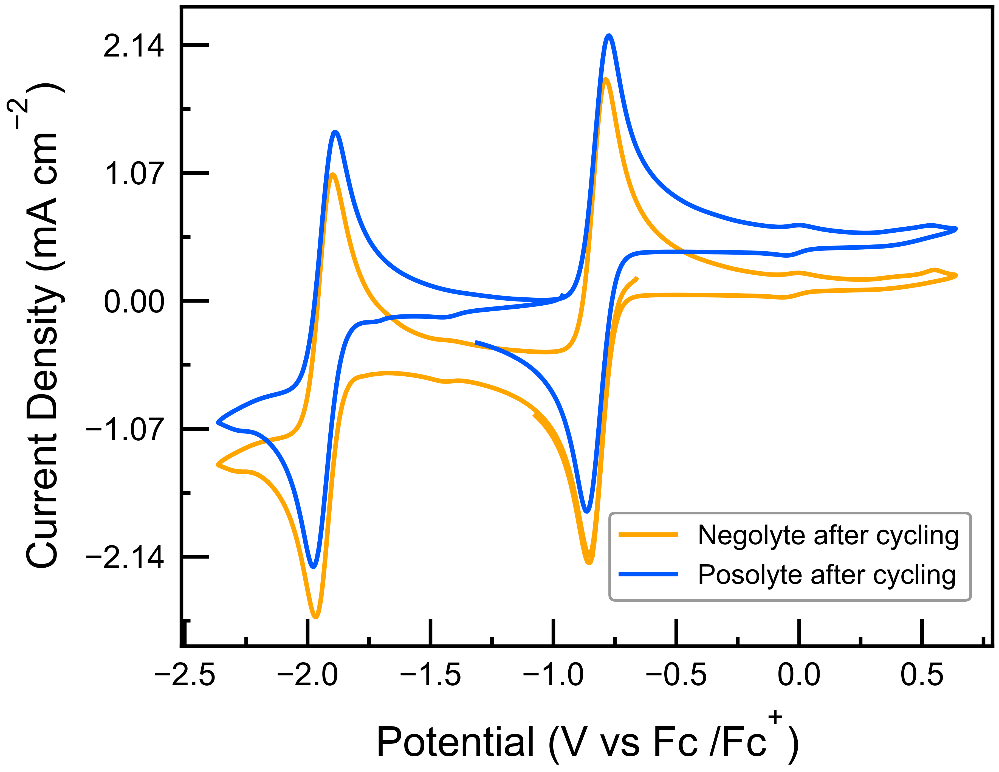


**Figure S19.** Cyclic voltammogram of the post-cycling solutions of symmetrical half-cell battery with 10 mM of a 50% SOC solution of **1** in 0.3 M [Bu_4_N][PF_6_]/MeCN.

**Figure S20.** ^1^H-NMR spectra of **1** in CD_3_CN (black) and of the post-half-cell-cycling of the negolyte (orange) and posolyte (blue) of a symmetrical H-cell battery with 10 mM of a 50% SOC solution of **1** in 0.3 M [Bu_4_N][PF_6_]/MeCN.

.

**Figure S21.** ^19^F-NMR spectra of **1** in CD_3_CN (black) and of the post-half-cell-cycling of the negolyte (orange) and posolyte (blue) of a symmetrical H-cell battery with 10 mM of a 50% SOC solution of **1** in 0.3 M [Bu_4_N][PF_6_]/MeCN.



**Figure S22.** (left) Charge and discharge voltage curves for the first 5 cycles of a H-cell battery with 5 mM of **1** (negolyte) and 10 mM of Fc (posolyte) in 0.3 M [Bu_4_N][PF_6_]/MeCN (cutoff voltages of 2.4 V and 0.1 V for charging and discharging, respectively, current ±0.8 mA). (right) discharging capacities (theoretical capacity of 0.67 mAh) and coulombic efficiency for each cycle. Total capacity fade of 85% after 100 cycles.


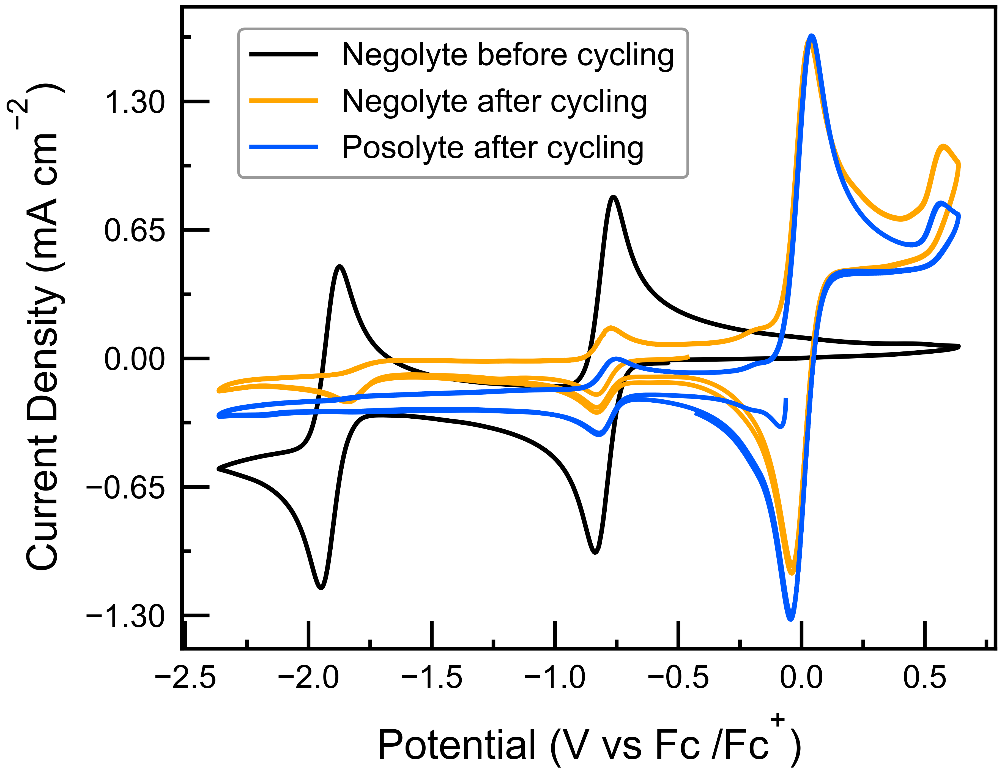


**Figure S23.** Cyclic voltammogram of the negolyte solution before two-electron H-cell cycling with **1** (negolyte) and 10 mM of Fc (posolyte) in 0.3 M [Bu_4_N][PF_6_]/MeCN and of the post-cycling solutions. From the peak currents obtained from cyclic voltammetry, capacity fade caused by crossover was estimated to be 20%.

**Figure S24.**  ^1^H-NMR spectra of **1** in CD_3_CN (black) and of the post-two-electron-cycling of the negolyte (orange) and posolyte (blue) of a battery with a 5 mM solution of complex **1** (Negolyte) and a 10 mM solution of ferrocene (Posolyte) in 0.3 M [Bu_4_N][PF_6_]/MeCN.

**Figure S25.**  ^19^F-NMR spectra of **1** in CD_3_CN (black) and of the post-two-electron-cycling of the negolyte (orange) and posolyte (blue) of a battery with a 5 mM solution of complex **1** (Negolyte) and a 10 mM solution of ferrocene (Posolyte) in 0.3 M [Bu_4_N][PF_6_]/MeCN.



**Figure S26.** (left) Charge and discharge voltage curves for the first 5 cycles of a H-cell battery with 5 mM of **2** (negolyte) and 10 mM of Fc (posolyte) in 0.3 M [Bu_4_N][PF_6_]/MeCN (cutoff voltages of 1.3 V and 0.2 V for charging and discharging, respectively, current ±0.8 mA). (right) discharging capacities theoretical capacity of 0.67 mAh) and coulombic efficiency for each cycle. Total capacity fade of 52% after 150 cycles.


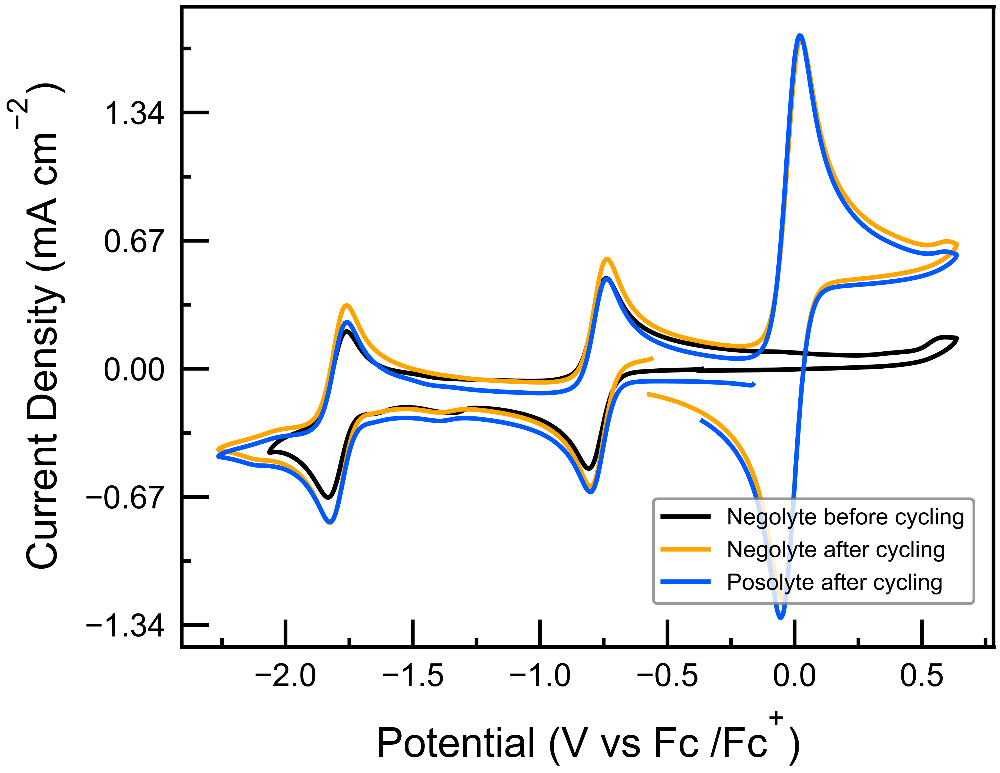


**Figure S27.** Cyclic voltammogram of the negolyte solution before one-electron cycling with **2** (negolyte) and 10 mM of Fc (posolyte) in 0.3 M [Bu_4_N][PF_6_]/MeCN and of the post-cycling solutions.

**Figure S28.**  ^1^H-NMR spectra of **2** in CD_3_CN (black) and of the post-one-electron-cycling of the negolyte (orange) and posolyte (blue) of a battery with a 5 mM solution of complex **2** (Negolyte) and a 10 mM solution of ferrocene (Posolyte) in 0.3 M [Bu_4_N][PF_6_]/MeCN.

**Figure S29.**  ^19^F-NMR spectra of **2** in CD_3_CN (black) and of the post-one-electron-cycling of the negolyte (orange) and posolyte (blue) of a battery with a 5 mM solution of complex **2** (Negolyte) and a 10 mM solution of ferrocene (Posolyte) in 0.3 M [Bu_4_N][PF_6_]/MeCN.



**Figure S30.** (left) Charge and discharge voltage curves for the first 5 cycles of a H-cell battery with 5 mM of **2** (negolyte) and 10 mM of Fc (posolyte) in 0.3 M [Bu_4_N][PF_6_]/MeCN (cutoff voltages of 2.4 V and 0.2 V for charging and discharging, respectively, current ±0.8 mA). (right) discharging capacities theoretical capacity of 1.34 mAh) and coulombic efficiency for each cycle. Total capacity fade of 50% after 75 cycles.


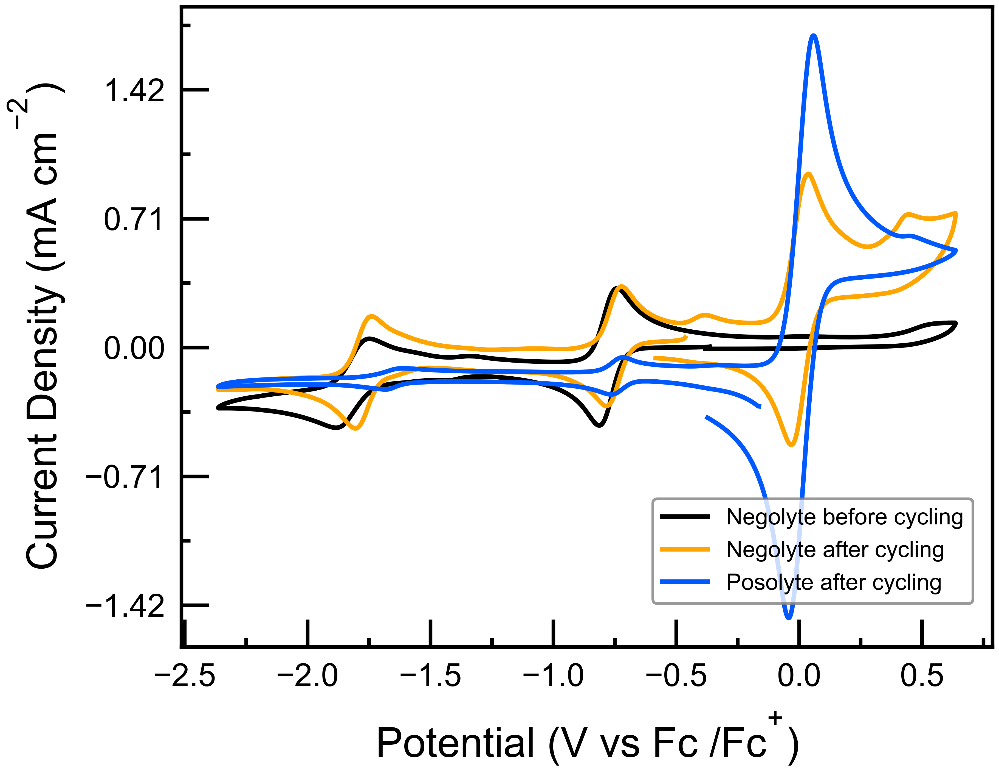


**Figure S31.** Cyclic voltammogram of the negolyte solution before two-electron cycling with **2** (negolyte) and 10 mM of Fc (posolyte) in 0.3 M [Bu_4_N][PF_6_]/MeCN and of the post-cycling solutions. From the peak currents obtained from cyclic voltammetry, capacity fade caused by crossover was estimated to be 17%.

**Figure S32.**  ^1^H-NMR spectra of **2** in CD_3_CN (black) and of the post-two-electron-cycling of the negolyte (orange) and posolyte (blue) of a battery with a 5 mM solution of complex **2** (Negolyte) and a 10 mM solution of ferrocene (Posolyte) in 0.3 M [Bu_4_N][PF_6_]/MeCN.

**Figure S33.**  ^19^F-NMR spectra of **2** in CD_3_CN (black) and of the post-two-electron-cycling of the negolyte (orange) and posolyte (blue) of a battery with a 5 mM solution of complex **2** (Negolyte) and a 10 mM solution of ferrocene (Posolyte) in 0.3 M [Bu_4_N][PF_6_]/MeCN.



**Figure S34.**  (left) Charge and discharge voltage curves for the first 5 cycles of a H-cell battery with 5 mM of **3** (negolyte) and 10 mM of Fc (posolyte) in 0.3 M [Bu_4_N][PF_6_]/MeCN (cutoff voltages of 1.8 V and 0.2 V for charging and discharging, respectively, current ±0.8 mA). (right) discharging capacities theoretical capacity of 0.67 mAh) and coulombic efficiency for each cycle. Total capacity fade of 9% after 70 cycles.


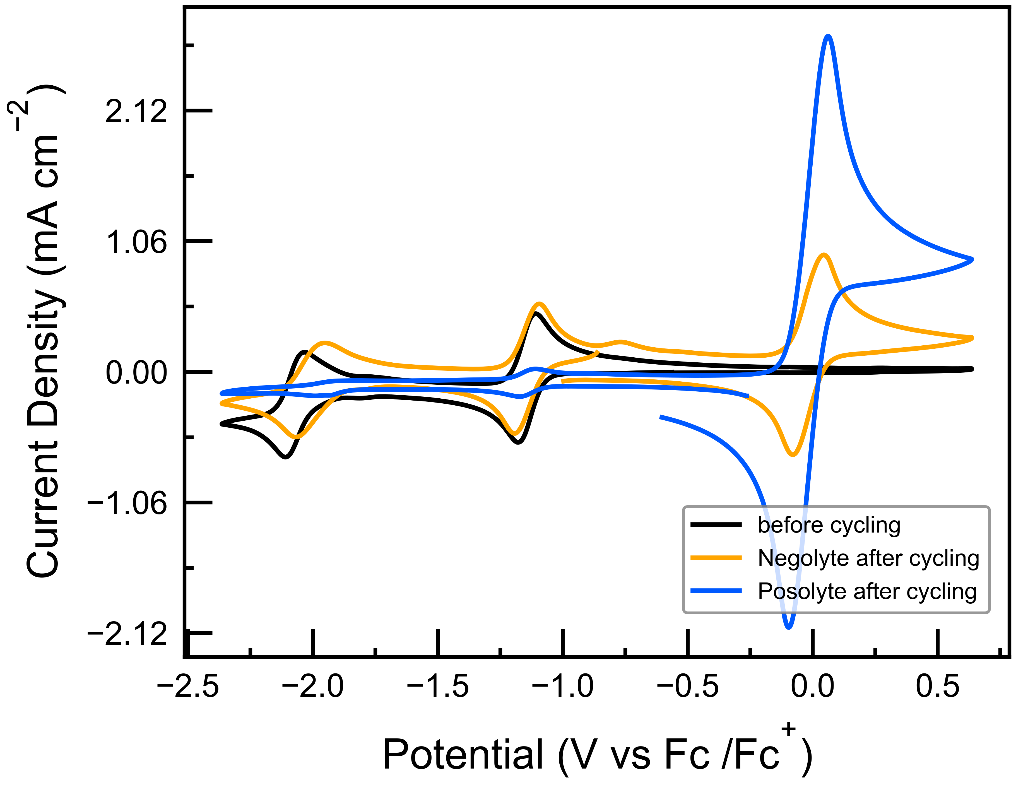


**Figure S35.**  Cyclic voltammogram of the negolyte solution before one-electron cycling with **3** (negolyte) and 10 mM of Fc (posolyte) in 0.3 M [Bu_4_N][PF_6_]/MeCN and of the post-cycling solutions. From the peak currents obtained from cyclic voltammetry, capacity fade caused by crossover was estimated to be 15%.

**Figure S36.**  ^1^H-NMR spectra of **3** in CD_3_CN (black) and of the post-one-electron-cycling of the negolyte (orange) and posolyte (blue) of a battery with a 5 mM solution of complex **3** (Negolyte) and a 10 mM solution of ferrocene (Posolyte) in 0.3 M [Bu_4_N][PF_6_]/MeCN.



**Figure S37.**  (left) Charge and discharge voltage curves for the first 5 cycles of a H-cell battery with 5 mM of **3** (negolyte) and 10 mM of Fc (posolyte) in 0.3 M [Bu_4_N][PF_6_]/MeCN (cutoff voltages of 2.4 V and 0.5 V for charging and discharging, respectively, current ±0.8 mA). (right) discharging capacities theoretical capacity of 1.34 mAh) and coulombic efficiency for each cycle. Total capacity fade of 52% after 75 cycles.


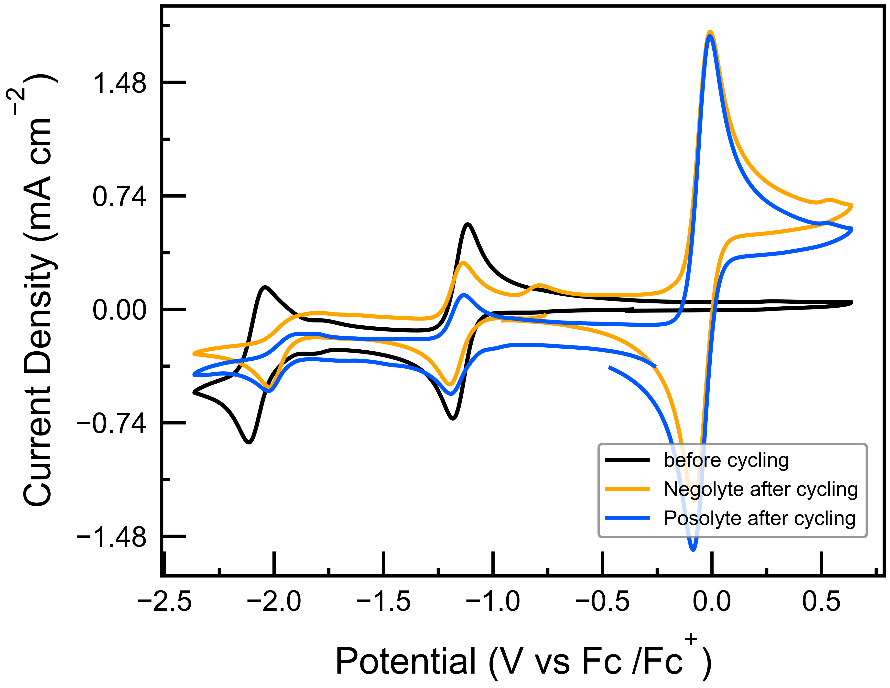


**Figure S38.**  Cyclic voltammogram of the negolyte solution before two-electron cycling with **3** (negolyte) and 10 mM of Fc (posolyte) in 0.3 M [Bu_4_N][PF_6_]/MeCN and of the post-cycling solutions. From the peak currents obtained from cyclic voltammetry, capacity fade caused by crossover was estimated to be 36%.

**Figure S39.** ^1^H-NMR spectra of **3** in CD_3_CN (black) and of the post-two-electron-cycling of the negolyte (orange) and posolyte (blue) of a battery with a 5 mM solution of complex **3** (Negolyte) and a 10 mM solution of ferrocene (Posolyte) in 0.3 M [Bu_4_N][PF_6_]/MeCN.



**Figure S40.** (left) Charge and discharge voltage curves for the first 5 cycles of a symmetrical H-cell battery with 5 mM of **3^-^** in both compartments in 0.3 M [Bu_4_N][PF_6_]/MeCN (cutoff voltages of 1.4 V and 0.1 V for charging and discharging, respectively, current ±0.8 mA). (right) discharging capacities (theoretical capacity of 0.67 mAh) and coulombic efficiency for each cycle. Total capacity fade of 40% after 100 cycles.


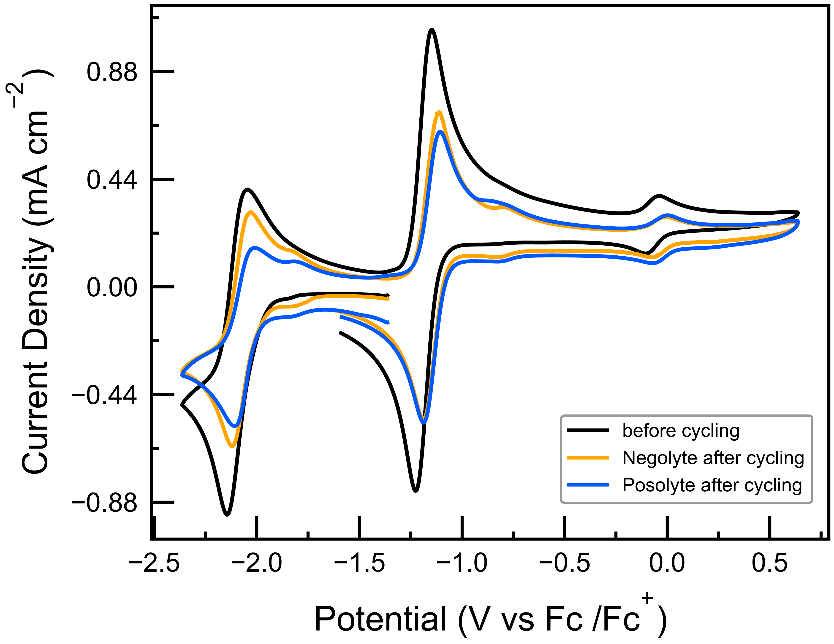


**Figure S41.**  Cyclic voltammogram of the negolyte solution before symmetric battery cycling with 5 mM of **3^-^** in 0.3 M [Bu_4_N][PF_6_]/MeCN and of the post-cycling solutions.

**Figure S42.** ^1^H-NMR spectra of **3** in CD_3_CN (black) and of the post-cycling of the negolyte (orange) and posolyte (blue) of a symmetric battery with a 5 mM solution of complex **3^-^** in both compartments in 0.3 M [Bu_4_N][PF_6_]/MeCN.

# Redox flow battery testing

**

**

**Figure S43.** Cycling performance of a redox flow battery with 0.20 mM **3^-^** in 0.3 M [Bu_4_N][PF_6_]/MeCN as both negolyte and posolyte (6 mL in each compartment) with polarity inversion after 285 cycles. (top left) Voltage versus time curve for the first 5 cycles. (top right) Charge and discharge capacity (82% capacity utilization reached in first cycle of the theoretical capacity of 3.2 mAh, and CE, VE, and EE for each cycle. (bottom left) Potential electrochemical impedance spectroscopy (PEIS) before cycling (at 0% SOC). (bottom right) I-V polarization and power density at 50% SOC.

# Determination of diffusion coefficients and standard rate constants by cyclic voltammetry

The diffusion coefficients were determined by performing cyclic voltammetry (CV) measurements recorded at scan rates between 10 to 500 mV s⁻¹. A plot of the cathodic and anodic peak currents against the square root of the scan rate yielded a linear relationship for all complexes, indicative of a diffusion-controlled redox process. The slope of this linear correlation was used in the Randles–Ševčík equation (E2) to calculate the diffusion coefficients.

$i_{p}=0.4463 nFAC\sqrt{\frac{nFvD}{RT}} (E2)$

where 𝑖_𝑝_ is the peak current (A), 𝑛 is the number of electrons transferred, 𝐹 is the Faraday constant (C mol⁻¹), 𝐴 is the electrode area (cm²), 𝐶 is the concentration of electroactive species (mol cm⁻³), 𝐷 is the diffusion coefficient (cm² s⁻¹), 𝑣 is the scan rate (V s⁻¹), 𝑅 is the gas constant (J mol⁻¹ K⁻¹), and 𝑇 is the absolute temperature (K).

Heterogeneous electron-transfer rate constants (𝑘^0^ ) were obtained using the Nicholson method.^[55]^ In this approach, the peak separation (Δ𝐸𝑝) between the anodic and cathodic waves is scan-rate dependent. These Δ𝐸𝑝 values were measured over the scan rate range, converted into the dimensionless kinetic parameter Ψ using Nicholson’s working curve, and plotted against the inverse of the scan rate. The slope of the resulting linear fit was then used to determine 𝑘^0^ according to E3.

$\Psi= \frac{\gamma k^{0}}{\sqrt{\pi nFvD/RT}} (E3)$

Where 𝛾=𝐷/𝐷′ represents the ratio of the diffusion coefficients for the reduction and re-oxidation processes.



**Figure S44.** (left) Cyclic voltammograms of 2.5 mM solution of **1** in 0.1 M [NBu_4_][PF_6_]/MeCN at varying scan rates. (right) Peak currents (ipc and ipa) from Figure S42 (left) vs. 𝑣 ^1/2^ used to determine the diffusion coefficients. (bottom) Ψ vs 𝑣 ^-1/2^  used to determine the electron transfer rate constants k^0^.



**Figure S45.** (left) Cyclic voltammograms of 2.5 mM solution of **2** in 0.1 M [NBu_4_][PF_6_]/MeCN at varying scan rates. (right) Peak currents (ipc and ipa) from Figure S42 (left) vs. 𝑣 ^1/2^ used to determine the diffusion coefficients. (bottom) Ψ vs 𝑣 ^-1/2^  used to determine the electron transfer rate constants k^0^.



**Figure S46.** (left) Cyclic voltammograms of 2.5 mM solution of **3** in 0.1 M [NBu_4_][PF_6_]/MeCN at varying scan rates. (right) Peak currents (ipc and ipa) from Figure S42 (left) vs. 𝑣 ^1/2^ used to determine the diffusion coefficients. (bottom) Ψ vs 𝑣 ^-1/2^  used to determine the electron transfer rate constants k^0^.

# Single Crystal Structure Analysis

**Experimental for Complex 3**

*Data Collection and Processing*. The sample was mounted on a nylon loop with a small amount of Paratone N oil. All X-ray measurements were made on a XtaLAB Synergy, Dualflex, HyPix-Arc 100 diffractometer at a temperature of 110.00(10) K. The unit cell dimensions were determined from a symmetry constrained fit of 38027 reflections with 4.84° < 2θ < 156.222°. The data collection strategy was a number of ω scans which collected data up to 157.158° (2θ). The frame integration was performed using CrysAlisPro (version 1.171.44.118a).^[56]^ The data were absorption corrected using a numerical absorption correction based on gaussian integration over a multifaceted crystal model. Scaling and an empirical absorption correction based on spherical harmonics were applied to the data using the SCALE3 ABSPACK algorithm as implemented in CrysAlisPro (version 1.171.44.118a).^[56]^

*Structure Solution and Refinement*. The structure was solved by using a dual space methodology using the SHELXT program.^[57]^ All non-hydrogen atoms were obtained from the initial solution. The hydrogen atoms were introduced at idealized positions and were allowed to refine isotropically. The structural model was fit to the data using full matrix least-squares based on *F^2^*. The calculated structure factors included corrections for anomalous dispersion from the usual tabulation. The structure was refined using the SHELXL program from the SHELX suite of crystallographic software.^[58]^ Graphic plots were produced using the Mercury program.^[59]^ Additional information and other relevant literature references can be found in the reference section of this website (http://xray.chem.uwo.ca).

**
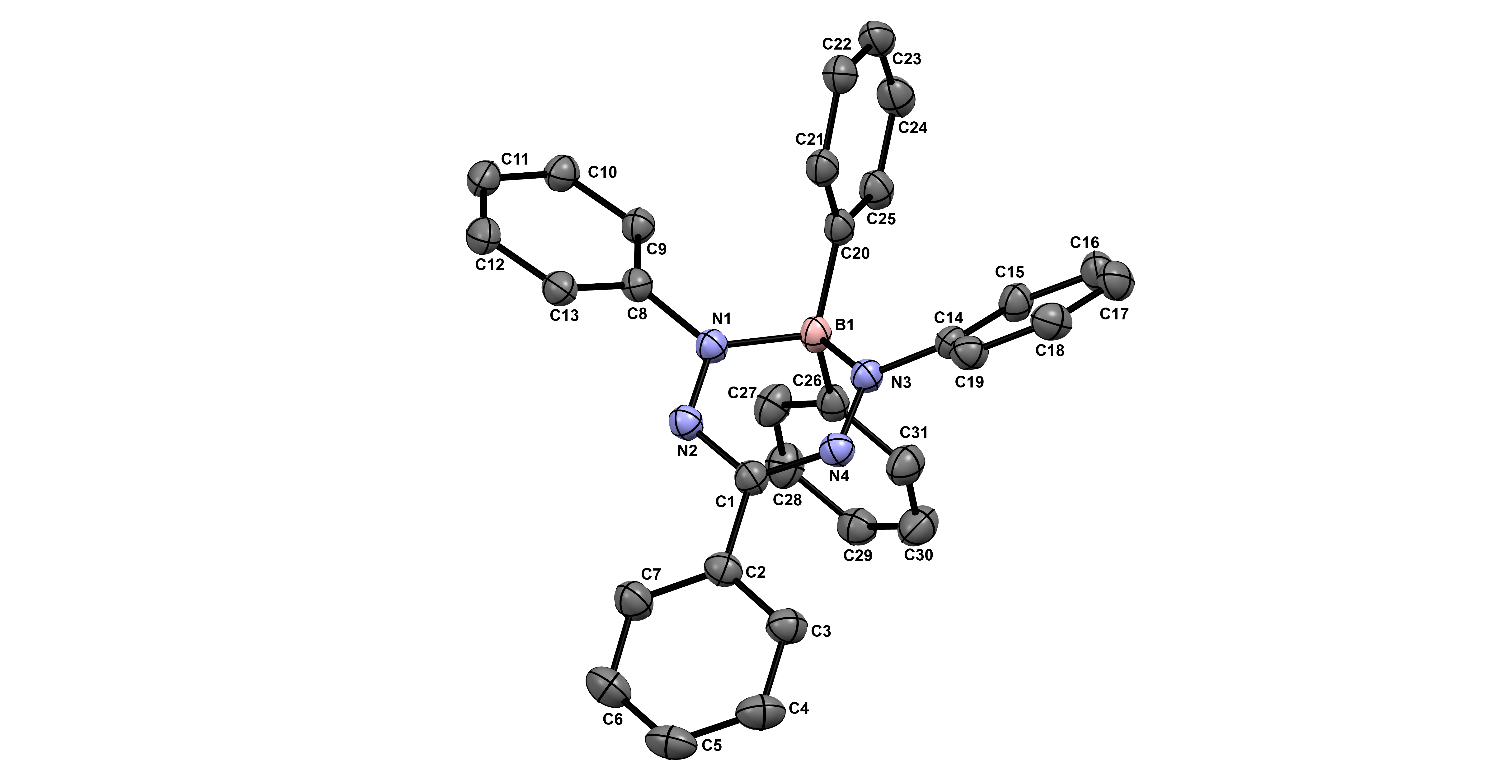
**

**Figure S47**. Solid state structure of complex **3** showing naming and numbering scheme. Ellipsoids are at the 50% probability level and hydrogen atoms were omitted for clarity.

***Table S1*. Summary of Crystal Data for *complex 3***

| Formula | C_31_H_25_BN_4_ |
| --- | --- |
| Formula Weight (*g/mol*) | 464.36 |
| Crystal Dimensions (*mm* ) | 0.159 × 0.113 × 0.020 |
| Crystal Colour and Habit | clear dark red plate |
| Crystal System | monoclinic |
| Space Group | P 2_1_/c |
| Temperature, K | 110.00(10) |
| *a*, Å | 9.66352(11) |
| *b*, Å | 13.93717(17) |
| *c*, Å | 18.2007(2) |
| α,° | 90 |
| β,° | 96.5317(11) |
| γ,° | 90 |
| V, Å^3^ | 2435.40(5) |
| Number of reflections to determine final unit cell | 38027 |
| Min and Max 2θ for cell determination, ° | 4.84, 156.222 |
| Z | 4 |
| F(000) | 976 |
| ρ (*g/cm^3^*) | 1.266 |
| λ, Å, (CuKα) | 1.54184 |
| μ, (*cm^-1^*) | 0.581 |
| Diffractometer Type | XtaLAB Synergy, Dualflex, HyPix-Arc 100 |
| Scan Type(s) | ω scans |
| Max 2θ for data collection, ° | 157.158 |
| Measured fraction of data | 1.000 |
| Number of reflections measured | 84195 |
| Unique reflections measured | 5231 |
| R_merge_ | 0.0269 |
| Number of reflections included in refinement | 5231 |
| Cut off Threshold Expression | I > 2σ(I) |
| Structure refined using | full matrix least-squares using F^2^ |
| Weighting Scheme | w=1/[σ^2^(Fo^2^)+(0.0487P)^2^+0.7493P] where P=(Fo^2^+2Fc^2^)/3 |
| Number of parameters in least-squares | 425 |
| R_1_ | 0.0371 |
| wR_2_ | 0.0958 |
| R_1_ (all data) | 0.0394 |
| wR_2_ (all data) | 0.0978 |
| GOF | 1.033 |
| Maximum shift/error | 0.001 |
| Min & Max peak heights on final ΔF Map (*e^-^*/Å) | -0.186, 0.436 |

Where:

R_1_ = *Σ* | |F_o_| - |F_c_| | / *Σ* F_o_

wR_2_ = [ *Σ*( *w*( F_o_^2^ - F_c_^2^ )^2^ ) / *Σ*(*w* F_o_^4^ ) ]^½^

GOF = [ *Σ*( *w*( F_o_^2^ - F_c_^2^ )^2^ ) / (No. of reflns. - No. of params. ) ]^½^

# References

[52] J. B. Gilroy, S. D. J. McKinnon, B. D. Koivisto, R. G. Hicks, “Electrochemical Studies of Verdazyl Radicals” *Org. Lett.* **2007**, 9, 4837–4840.

[53] S. M. Barbon, V. N. Staroverov, J. B. Gilroy, “Effect of Extended π Conjugation on the Spectroscopic and Electrochemical Properties of Boron Difluoride Formazanate Complexes” J. Org. Chem. 2015, 80, 5226–5235.

[54] J. D. Milshtein, A. P. Kaur, M. D. Casselman, J. A. Kowalski, S. Modekruti, P. L. Zhang, N. H. Attanayake, C. F. Elliott, S. R. Parkin, C. Risko, F. R. Brushett, S. A. Odom, “High Current Density, Long Duration Cycling of Soluble Organic Active Species for Non-Aqueous Redox Flow Batteries*” Energy Environ. Sci.* **2016**, 9, 3531–3543.

[55] R. S. Nicholson, “Theory and Application of Cyclic Voltammetry for Measurement of Electrode Reaction Kinetics” *Anal. Chem*. **1965**, 37, 1351–1355.

[56] Rigaku Oxford Diffraction, CrysAlisPro Software System, Version 1.171.44.118a, Rigaku Corporation, Wroclaw (Poland), **2025**.

[57] G. M. Sheldrick, *Acta Crystallogr.* **2015**, A71, 3–8.

[58] G. M. Sheldrick*, Acta Crystallogr.* **2015**, C71, 3–8.

[59] C. F. Macrae, I. J. Bruno, J. A. Chisholm, P. R. Edington, P. McCabe, E. Pidcock, L. Rodriguez Monge, R. Taylor, J. van de Streek, P. A. Wood, “Mercury CSD 2.0–new features for the visualization and investigation of crystal structures” *J. Appl. Crystallogr.* **2008**, 41, 466–470.
